# Supplementary material for: Early head-up mobilisation versus standard care for patients with severe acquired brain injury: A systematic review with meta-analysis and Trial Sequential Analysis
Source: PLoS One. 2020 Aug 13;15(8):e0237136. doi: 10.1371/journal.pone.0237136 (PMC7425882; doi:10.1371/journal.pone.0237136)
Supplement: S1 File — (PDF) [file pone.0237136.s001.pdf]

Supporting material for

Early head-up mobilisation versus standard care for patients with severe acquired brain injury: a systematic review with meta-analysis and Trial Sequential Analysis

Christian G Riberholt, Vibeke Wagner, Jane Lindschou, Christian Gluud, Jesper Mehlsen, Kirsten Møller

Table of contents

- **Protocol (PROSPERO - CRD42018088790)**
- **Checklist (PRISMA)**
- **Search strategy (Medline)**
- **Table 1. Characteristics of excluded studies and trials**
- **Fig 1 to 13. Trial Sequential Analysis.**
- **Table 2. Number of patients with at least one individualised serious adverse event or adverse event not considered serious sorted according to total number of patients with event.**

Early head up mobilisation for patients with severe acquired brain injury

Christian Riberholt, Vibeke Wagner, Jane Lindschou, Christian Gluud, Jesper Mehlsen, Kirsten Møller

Citation

Christian Riberholt, Vibeke Wagner, Jane Lindschou, Christian Gluud, Jesper Mehlsen, Kirsten Møller. Early head up mobilisation for patients with severe acquired brain injury. PROSPERO 2018 CRD42018088790 Available from:
https://www.crd.york.ac.uk/prospero/display_record.php?ID=CRD42018088790

Review question

How does early head up mobilisation with head and torso elevated more than 60 degrees affect mortality or poor functional outcome compared to standard care in patients with severe brain injury?

Searches

We will search the following bibliographic databases: MEDLINE (Ovid); Cochrane Central Register of Controlled Trials (CENTRAL, The Cochrane Library); EMBASE (Ovid); CINAHL (EBSCO); PsycINFO; Science Citation Index Expanded on Web of Science; and PEDro.

The search strategy will include terms relating to or describing the conditions and the intervention.The search strategy for MEDLINE (the preliminary) will be adapted for searches in the other databases. We will apply the Cochrane sensitivity-maximising randomised clinical trial filter (Lefebvre 2011) to MEDLINE (Ovid) and adaptations of it to the other databases, except CENTRAL.

References of previous reviews and included papers will be screened for further relevant trials.
The authors of included trials will be contacted to seek information about relevant published and unpublished trials.

Furthermore, we will search for ongoing and unidentified randomised clinical trials on:

Google Scholar; Database on Research in Stroke (DORIS); The Turning Research into Practice (TRIP) Database; ClinicalTrials.gov; EU Clinical Trial Register; Chinese Clinical Trial Registry (ChCTR); International Standard Randomised Controlled Trial Number (ISRCTN) registry; Pan African Clinical Trials Registry (PACTR); Australian New Zealand Clinical Trials Registry (ANZCTR); Clinical Trials Registry - India (CTRI); and the World Health Organization (WHO) International Clinical Trials Registry Platform (ICTRP) search portal.

The search strategy for MEDLINE will be available in the published protocol. There will be no language restrictions or restrictions due to publication date.

Types of study to be included

We will include randomised clinical trials aiming at evaluating benefits and harms of early head-up mobilisation irrespective of language, publication date, publication type, or publication status. We will not directly search for quasi-randomised clinical trials or observational studies, but valid studies will be included for the analysis of harms if we should encounter such studies during our searches. We are aware that our selection of designs may be a limitation to our review in a way that it will limit our chances of discovering harms, especially long term and very rare harms resulting from the intervention.

Condition or domain being studied

Severe acquired brain injury described broadly as brain damage that occurs after birth, and which is not related to congenital or degenerative conditions. Early head-up mobilisation to more than 60 degrees over horizontal.

Participants/population

Inclusion:

Major stroke: Interruption of blood supply to the brain usually because of one or more bursting blood vessels (haemorrhagic) or because of blockage of one or more vessels (ischaemic) (Endres 2009), associated with a National Institute of Health Stroke Scale (NIHSS) score >16 (Bernhardt 2015);
Severe traumatic brain injury: Injury resulting from trauma to the head and its direct consequences, including hypoxia, hypotension, intracranial haemorrhage and raised intracranial pressure (Hawryluk 2015), with a duration of post-traumatic amnesia of more than 28 days;
Severe diffuse brain injury: Diffuse damage arising from trauma due to a range of other acute incidents including hypoxia (e.g. resulting from drowning, electrocution, anaesthetic accident) (Hawryluk 2015), with a duration of post-injury amnesia of more than 28 days.

Intervention(s), exposure(s)

Any type of intervention with a direct focus on head-up mobilisation.
For the purpose of this review, we consider head-up mobilisation as any intervention during which the patient’s head and torso are elevated at an angle of at least 60 degrees relative to the horizontal (with a minimal clinical difference of -10 degrees) for at least 10 minutes, whether or not the legs are placed horizontally or below this line. Interventions could be carried out by elevating the head of the bed to more then 60 degrees, mobilising to a seated position on the edge of the bed (with support if needed) or a wheel chair, or using a tilt table or other mechanical device to attain a standing position.

We will accept any form of co-intervention as long as it is equally distributed between the intervention group and the control group. We will check for co-intervention after randomisation in both intervention groups and consider any differences in our conclusion.

Comparator(s)/control

Any intervention enforcing elevations less than 50 degrees in the treatment plan or standard care not directly emphasizing head and torso elevation or mobilisation out of bed.

Context

Studies in hospital intensive care units, stroke units or other relevant units which may treat the above mentioned participants.

Main outcome(s)

Mortality or poor functional outcome. An example of poor functional outcome for patients with stroke could be a modified Rankin Scale (Swieten 1988) score above 4 (5 indicating severe disability and 6 indicating death) or for patients with traumatic brain injury could be Global Outcome Scale extended (Wilson 1998) score below 5 (4 indicating upper severe disability, 3 lower severe disability, 2 vegetative state and 1 death).

Quality of life measured on any scale validated for measuring quality of life.

Serious adverse events. A serious adverse event is defined as any untoward medical complication that resulted in death; was life threatening; required hospitalisation or prolongation of existing hospitalisation; resulted in persistent or significant disability; or jeopardised the patient (ICH-GCP 1997).

Timing and effect measures

We will assess all outcomes at the end of intervention (as is defined by the trials) as the primary outcome of this review and at longest follow-up, if possible.

Additional outcome(s)

Non-serious adverse events: A non-serious adverse event is defined as any untoward medical complication that does not result in death; was not life threatening; did not required hospitalisation or prolongation of existing hospitalisation; did not result in persistent or significant disability; or did not jeopardise the patient (ICH-GCP 1997).

Level of consciousness as measured by the Coma Recovery Scale-Revised (Giacino 2004) or other relevant scales as defined by the individual trialist.

Timing and effect measures

We will assess all outcomes at the end of intervention (as is defined by the trials) as the primary outcome of this review and at longest follow-up, if possible.

Data extraction (selection and coding)

Three review authors (CR, VW, and JM) will extract data in an independent manner from included trials fulfilling the inclusion criteria. If disagreement occurs, this will be solved by discussion. If agreement is not possible, KM, JL or CG will arbitrate. If data on patient or trial characteristics or outcome measures are not described in the published reports, we will write to the study authors to obtain missing information or if information is not sufficiently described in the studies.

The following data will be extracted:

General information: publication status, title, authors' names, source, country, contact address, language of publication, year of publication, duplicate publication.

Trial characteristics: design and setting.

Interventions: type of intervention used for mobilisation, dose, duration, type of control intervention.

Participants: inclusion and exclusion criteria, number of participants randomised in intervention and control groups, participant demographics such as sex and age, and baseline characteristics for patients relevant for subgroup analysis.

Outcomes: Number of patients analysed for each outcome. Please see Primary and secondary outcomes measures above.

Risk of bias: please see Risk of bias (quality) assessment below.

Data relevant for subgroup and sensitivity analyses (Please see Analysis of subgroups or subsets).

Risk of bias (quality) assessment

Risk of bias will be assessed by three independent authors (CR, VW, and JM) using the criteria as described in the Cochrane Handbook for Systematic Reviews of Interventions (Higgins 2011).

Random sequence generation

- Low risk: If sequence generation was achieved using computer random number generator or a random number table. Drawing lots, tossing a coin, shuffling cards, and throwing dice were also considered adequate if performed by an independent adjudicator.

- Unclear risk: If the method of randomisation was not specified, but the trial was still presented as being randomised.

- High risk: If the allocation sequence is not randomised or only quasi-randomised. These trials will only be included for assessment of harms.

Allocation concealment

- Low risk: If the allocation of patients was performed by a central independent unit, on-site locked computer or identical-looking numbered sealed envelopes.

- Uncertain risk: If the trial was classified as randomised but the allocation concealment process was not described.

- High risk: If the allocation sequence was familiar to the investigators who assigned the participants.

Blinding of participants and treatment providers

- Low risk: If the participants and the treatment providers were blinded to intervention allocation and this was described.

- Uncertain risk: If the procedure of blinding was insufficiently described.

- High risk: If blinding of participants and treatment providers was not performed.

Blinding of outcome assessor

- Low risk: If it was mentioned that outcome assessors were blinded and this was described.

- Uncertain risk: If it was not mentioned if the outcome assessors in the trial were blinded or the extent of blinding was insufficiently described.

- High risk: If blinding or incomplete blinding of outcome assessors was performed.

Incomplete outcome data

- Low risk: If missing data were unlikely to make treatment effects depart from plausible values. This could be either (1) there were no drop-outs or withdrawals for all outcomes, or (2) the numbers and reasons for the withdrawals and drop-outs for all outcomes were clearly stated and could be described as being similar to both groups. Generally, the trial is judged as at a low risk of bias due to incomplete outcome data if drop-outs are less than 5%. However, the 5% cut-off is not definitive.

- Uncertain risk: If there was insufficient information to assess whether missing data were likely to induce bias on the results.

- High risk: If the results were likely to be biased due to missing data either because the pattern of drop-outs could be described as being different in the two intervention groups or the trial used improper methods in dealing with missing data (e.g. last observation carried forward).

Selective outcome reporting

- Low risk of bias: If a protocol was published before or at the time the trial was begun and the outcomes specified in the protocol were reported on. If there is no protocol or the protocol was published after the trial has begun, reporting of all-cause mortality and serious adverse events will grant the trial a grade of low risk of bias.

- Uncertain risk of bias: If no protocol was published and the outcome all-cause mortality and serious adverse events were not reported on.

- High risk of bias: If the outcomes specified in the protocol were not reported on.

Other bias

- Low risk of bias: If the trial appears to be free of other components (for example, academic bias or for-profit bias) that could put it at risk of bias.

- Unclear risk of bias: If the trial may or may not be free of other components that could put it at risk of bias.

- High risk of bias: If there are other factors in the trial that could put it at risk of bias (for example, authors conducted trials on the same topic, for-profit bias, etc.).

Overall risk of bias

- Low risk of bias: The trial will be classified as overall "low risk of bias" only if all of the bias domains described in the above paragraphs are classified as "low risk of bias".

- High risk of bias: The trial will be classified as "high risk of bias" if any of the bias risk domains described above are classified as "unclear" or "high risk of bias".

We will assess the domains "blinding of outcome assessment", "incomplete outcome data", and "selective outcome reporting" for each outcome result. Thus, we can assess the bias risk for each outcome assessed in addition to each trial. Our primary conclusion will be based on the results of our primary outcome results with overall low risk of bias.

Strategy for data synthesis

Measures of treatment effect

We will analyse dichotomous data as risk ratios with 95% confidence intervals (CIs) and continuous data as mean difference or standardised mean difference with 95% CIs. For dichotomous and continuous data we will apply Trial Sequential Analysis-adjusted CI (please see Data synthesis below).

We will give a narrative description of skewed data reported as medians and interquartile ranges.

Unit of analysis issues

We will include randomised clinical trials. For trials using cross-over design, we will only include data from the first comparison period (Elbourne 2002; Deeks 2017).

Cluster trials

Studies increasingly employ ’cluster randomisation’ (such as randomisation by clinician or practice), but the analysis and pooling of clustered data poses problems. First, authors often fail to account for intraclass correlation (ICC) in clustered studies, leading to a ’unit of analysis’ error (Divine 1992), whereby P values may become spuriously low, CIs unduly narrow, and statistical significance overestimated. This causes type I errors (Bland 1997; Gulliford 1999).

For cluster-randomised trials, we will calculate the ‘design effect’. This is calculated using the mean number of participants per cluster (M) and the ICC [design effect = 1 + (M - 1) * ICC] (Donner 2002). Where clustering is not accounted for in primary studies, we will seek to contact the first authors of studies to obtain ICCs for their clustered data and to adjust for this by using accepted methods (Gulliford 1999). If the ICC is not reported or obtainable it will be assumed to be 0.1 (Ukoumunne 1999).

We will then calculate the ’effective sample size’. For dichotomous outcomes, the number or participants and the number experiencing the event should be divided by the design effect. For continuous outcomes, only the sample size needs to be divided by the design effect (Higgins 2011). After this, the numbers can be entered into RevMan.

Trials with multiple intervention groups

Where a trial involves more than two intervention groups, if relevant, the additional intervention groups will be presented in comparisons. If data are binary these will be simply added and combined within the two-by-two table. If data are continuous we will combine data following the formula in section 7.7.3.8 (Combining groups) of the Cochrane Handbook for Systematic Reviews of Interventions (Higgins 2011). Where the additional treatment arms are not relevant, these data will not be reproduced.

Dealing with missing data

We will contact corresponding authors of trials to obtain data and verify key study characteristics (i.e. for data extraction and for assessment of risk of bias). We will use previously recommended methods of imputation to evaluate the influence of missing data (Higgins 2008).

Assessment of heterogeneity

We will visually inspect forest plots (Jakobsen 2014) and will express heterogeneity as I^2 values, using a threshold of the Chi^2 test of $P < 0.10$ and the following thresholds (Higgins 2002; Higgins 2003):

0% to 40%: might not be important.30% to 60%: may represent moderate heterogeneity. 50% to 90%: may represent substantial heterogeneity. 75% to 100%: may represent considerable heterogeneity.

Assessment of reporting biases

As we expect few and small studies for this review, the assessment of publication bias does not seem possible. Otherwise, publication bias will be examined by funnel-plot.

Data synthesis

We will perform the analysis using the statistical programs Review Manager 5 (RevMan 2014 [Computer program]), STATA, and Trial Sequential Analysis (TSA 2011 [Computer program]). If the interventions, participants, and underlying questions are similar, we will undertake a meta-analysis using both a fixed-effect and a random-effects model, and report any differences. We will conduct meta-analysis using random-effects and fixed-effect models and will report the most conservative result, using a P value for the primary outcomes of 2.5% as significant (Jakobsen 2014).

For the continuous outcomes (quality of life scales and the Coma Recovery Scale-Revised) the minimal clinically relevant differences will be 0.5 standard deviation calculated from the observed variance of the studies.

Trial Sequential Analysis

Cumulative meta-analyses are at risk of producing random errors due to sparse data and/or multiple testing of accumulating data (Brok 2008; Brok 2009; Higgins 2011; Pogue 1997; Thorlund 2009 Wetterslev 2008; Wetterslev 2017). Trial Sequential Analysis (TSA),(TSA 2011 [Computer program]), can be applied to control random errors and to assess the risks of imprecision (http://www.ctu.dk/tsa/) (Jakobsen 2014; Thorlund 2011). Similar to a sample size calculation in a randomised clinical trial, TSA calculates the required information size for the meta-analysis (that is the number of participants needed in a meta-analysis to detect or reject a certain intervention effect) in order to minimise random errors (Wetterslev 2009; Wetterslev 2017). The required information size for a dichotomous outcome takes into account the event proportion in the control group, the assumption of a plausible risk ratio (RR) reduction, and the heterogeneity of the meta-analysis (Turner 2013; Wetterslev 2009). TSA enables testing for significance to be conducted each time a new trial is included in the meta-analysis. On the basis of the required information size, trial sequential monitoring boundaries can be constructed. This enables one to determine the statistical inference concerning cumulative meta-analysis that has not yet reached the required information size (Wetterslev 2008; Wetterslev 2017).

Firm evidence for benefit or harms may be established if the trial sequential monitoring boundary is crossed before reaching the required information size, in which case further trials may turn out to be superfluous. In contrast, if the boundary is not surpassed one may conclude that it is necessary to continue with further trials before a certain intervention effect can be detected or rejected. Firm evidence for lack of the postulated intervention effect can also be assessed with TSA. This occurs when the cumulative Z-score crosses the trial sequential monitoring boundaries for futility.

For dichotomous outcomes, we will estimate the required information size based on the proportion of patients with an outcome in the control group, a relative risk reduction of 20%, an alpha of 2.5% for primary outcomes, and 3.33% for secondary outcomes, a beta of 10%, and a variance suggested by the trials in a random-effects meta-analysis (diversity-adjusted required information size) (Jakobsen 2014; Kotecha 2016; Wetterslev 2009). In case there is some evidence of effect of the intervention, a supplementary TSA will use the limit of the CI closest to 1.00 as the anticipated intervention effect (Jakobsen 2014). Additionally, we will calculate TSA-adjusted CI.

For continuous outcomes, we have not identified valid previous data on effect sizes on quality of life so we have chosen to use SD/2 as anticipated intervention effect. Hence, we will estimate the required information size based on the SD observed in the control group of trials with low risk of bias or lower risk of bias and a minimal relevant difference of the observed SD/2, an alpha value of 2.5% for primary outcomes and 3.33% for secondary outcomes, a beta value of 10%, and a diversity suggested by the trials in the meta-analysis (Jakobsen 2014; Wetterslev 2009). In case there is some evidence of effect of the intervention, as a supplementary TSA will use the limit of the CI closest to 0.00 as the anticipated intervention effect (Jakobsen 2014). Additionally, we will calculate TSA-adjusted CI.

Analysis of subgroups or subsets

We plan to group the results of included studies according to the following methodological and clinical considerations:

- Trials at low risk of bias compared to trials at high risk of bias.

- Type of mobilisation intervention used (tilt table intervention compared to other interventions).

- According to type of injury (stroke patients, traumatic brain injury, or diffuse acquired brain injury).

- Duration of the intervention period (long duration is defined as more than 20 minutes per day for at least 14 days compared to less than 20 minutes or less than 14 days).

- Intensity of the intervention (high intensity is defined as more than one hour per day compared to one hour or less per day).

- Frequency of the intervention (high intensity frequency is defined as four or more intervention sessions per day during the intervention period compared to three or less per day).

- Timing of the intervention (start of intervention earlier than 48 hours after brain injury compared to later than 48 hours after the brain injury).

Contact details for further information

Christian Gunge Riberholt
christian.riberholt@regionh.dk

Organisational affiliation of the review

Department of Neurorehabilitation / TBI unit, Rigshospitalet

Review team members and their organisational affiliations

Mr Christian Riberholt. Department of Neurorehabilitation / TBI unit, Rigshospitalet, Copenhagen, Denmark
Ms Vibeke Wagner. Department of Neurorehabilitation / TBI unit, Rigshospitalet, Copenhagen, Denmark
Ms Jane Lindschou. The Copenhagen Trial Unit, Centre for Clinical Intervention Research, Rigshospitalet, Copenhagen, Denmark
Dr Christian Gluud. The Copenhagen Trial Unit, Centre for Clinical Intervention Research, Rigshospitalet, Copenhagen, Denmark
Dr Jesper Mehlsen. Syncope Centre, Department of Cardiology, Bispebjerg and Frederiksberg Hospital, Frederiksberg, Denmark.
Professor Kirsten Møller. Department of Neuroanaesthesiology, Rigshospitalet, University of Copenhagen, Copenhagen, Denmark

Type and method of review

Intervention, Meta-analysis, Systematic review

Anticipated or actual start date

01 May 2018

Anticipated completion date

31 December 2018

Funding sources/sponsors

Funding provided by The Danish Victims fund (16-910-00043)

Conflicts of interest

Language
(there is not an English language summary)

Country
Denmark

Stage of review
Review Ongoing

Subject index terms status
Subject indexing assigned by CRD

Subject index terms
Brain Injuries; Early Ambulation; Glasgow Coma Scale; Humans

Date of registration in PROSPERO
26 April 2018

Date of publication of this version
26 October 2018

Details of any existing review of the same topic by the same authors

Stage of review at time of this submission

Stage	Started	Completed
Preliminary searches	Yes	Yes
Piloting of the study selection process	Yes	Yes
Formal screening of search results against eligibility criteria	Yes	Yes
Data extraction	Yes	No
Risk of bias (quality) assessment	Yes	No
Data analysis	No	No

Versions
26 April 2018
26 October 2018

PROSPERO
This information has been provided by the named contact for this review. CRD has accepted this information in good faith and registered the review in PROSPERO. The registrant confirms that the information supplied for this submission is accurate and complete. CRD bears no responsibility or liability for the content of this registration record, any associated files or external websites.

PRISMA Checklist

Section/topic	#	Checklist item	Reported on page #
**TITLE**			
Title	1	Identify the report as a systematic review, meta-analysis, or both.	P 1
**ABSTRACT**			
Structured summary	2	Provide a structured summary including, as applicable: background; objectives; data sources; study eligibility criteria, participants, and interventions; study appraisal and synthesis methods; results; limitations; conclusions and implications of key findings; systematic review registration number.	P 2-3
**INTRODUCTION**			
Rationale	3	Describe the rationale for the review in the context of what is already known.	P 4-5
Objectives	4	Provide an explicit statement of questions being addressed with reference to participants, interventions, comparisons, outcomes, and study design (PICOS).	P 6
**METHODS**			
Protocol and registration	5	Indicate if a review protocol exists, if and where it can be accessed (e.g., Web address), and, if available, provide registration information including registration number.	P 6
Eligibility criteria	6	Specify study characteristics (e.g., PICOS, length of follow-up) and report characteristics (e.g., years considered, language, publication status) used as criteria for eligibility, giving rationale.	P 6-9
Information sources	7	Describe all information sources (e.g., databases with dates of coverage, contact with study authors to identify additional studies) in the search and date last searched.	P 9-10
Search	8	Present full electronic search strategy for at least one database, including any limits used, such that it could be repeated.	S1 File
Study selection	9	State the process for selecting studies (i.e., screening, eligibility, included in systematic review, and, if applicable, included in the meta-analysis).	P 11
Data collection process	10	Describe method of data extraction from reports (e.g., piloted forms, independently, in duplicate) and any processes for obtaining and confirming data from investigators.	P 11-12
Data items	11	List and define all variables for which data were sought (e.g., PICOS, funding sources) and any assumptions and simplifications made.	P 11-12
Risk of bias in individual studies	12	Describe methods used for assessing risk of bias of individual studies (including specification of whether this was done at the study or outcome level), and how this information is to be used in any data synthesis.	P 12-17
Summary	13	State the principal summary measures (e.g., risk ratio, difference in means).	P 14

measures			
Synthesis of results	14	Describe the methods of handling data and combining results of studies, if done, including measures of consistency (e.g., I^2) for each meta-analysis.	P 13-17
Risk of bias across studies	15	Specify any assessment of risk of bias that may affect the cumulative evidence (e.g., publication bias, selective reporting within studies).	P 12-13
Additional analyses	16	Describe methods of additional analyses (e.g., sensitivity or subgroup analyses, meta-regression), if done, indicating which were pre-specified.	P 14-17
**RESULTS**			
Study selection	17	Give numbers of studies screened, assessed for eligibility, and included in the review, with reasons for exclusions at each stage, ideally with a flow diagram.	Figure 1, 18-22
Study characteristics	18	For each study, present characteristics for which data were extracted (e.g., study size, PICOS, follow-up period) and provide the citations.	Table 1, p 19-21
Risk of bias within studies	19	Present data on risk of bias of each study and, if available, any outcome level assessment (see item 12).	Table 1, Figure 2, p 22-23
Results of individual studies	20	For all outcomes considered (benefits or harms), present, for each study: (a) simple summary data for each intervention group (b) effect estimates and confidence intervals, ideally with a forest plot.	Figure 4-16
Synthesis of results	21	Present results of each meta-analysis done, including confidence intervals and measures of consistency.	P 23-34
Risk of bias across studies	22	Present results of any assessment of risk of bias across studies (see Item 15).	Figure 3
Additional analysis	23	Give results of additional analyses, if done (e.g., sensitivity or subgroup analyses, meta-regression [see Item 16]).	P 20-28
**DISCUSSION**			
Summary of evidence	24	Summarize the main findings including the strength of evidence for each main outcome; consider their relevance to key groups (e.g., healthcare providers, users, and policy makers).	Table 3<br>P 34-35
Limitations	25	Discuss limitations at study and outcome level (e.g., risk of bias), and at review-level (e.g., incomplete retrieval of identified research, reporting bias).	P 37-39
Conclusions	26	Provide a general interpretation of the results in the context of other evidence, and implications for future research.	P 41
**FUNDING**			
Funding	27	Describe sources of funding for the systematic review and other support (e.g., supply of data); role of funders for the systematic review.	P 41-42

From: Moher D, Liberati A, Tetzlaff J, Altman DG, The PRISMA Group (2009). Preferred Reporting Items for Systematic Reviews and Meta-Analyses: The PRISMA Statement. PLoS Med 6(7): e1000097. doi:10.1371/journal.pmed1000097

Search history - MEDLINE Ovid SP (1946 to May 2018) (4116 hits)

1. exp Brain Diseases/

2. exp Craniocerebral Trauma/

3. (brain and (disease* or disorder* or injur* or lacerati* or accident* or hemorrhage*)).ti,ab.

4. (craniocerebral and (trauma* or injur*)).ti,ab.

5. (cerebrovascular and (disease* or disorder* or occlusion* or insufficienc* or accident* or apoplex* or stroke*)).ti,ab

6. (intracranial and (disease* or disorder* or aneurism* or hemorrhage*)).ti,ab.

7. (head and (trauma* or injur*)).ti,ab.

8. ((posterior fossa or subarachnoid) and hemorrhage*).ti,ab.

9. (encephalo* or apoplex* or cerebral stroke*).ti,ab.

10. 1 or 2 or 3 or 4 or 5 or 6 or 7 or 8 or 9

11. exp early ambulation/ or exp exercise therapy/ or exp neurological rehabilitation/

12. exp Posture/

13. ((early and (ambulation* or mobili* or rehab*)) or accelerated ambulation*).ti,ab.

14. (exercis* and (therap* or rehab* or remedial)).ti,ab.

15. ((neurologic* and rehab*) or neurorehab*).ti,ab.

16. (continuous passive and (motion or movement) and therap*).ti,ab.

17. ((position* and (seated or standing or sitting)) or posture* or head*up).ti,ab.

18. 11 or 12 or 13 or 14 or 15 or 16 or 17

19. 10 and 18

20. (random* or blind* or placebo* or meta-analys*).mp. [mp=title, abstract, original title, name of substance word, subject heading word, keyword heading word, protocol supplementary concept word, rare disease supplementary concept word, unique identifier, synonyms]

21. 19 and 20

22. limit 21 to humans

Table 1. Characteristics of excluded studies and trials

Study	Reason for exclusion
**Abdulwahab 1996** [1]	Wrong study design, no adverse events reported
**Abouzari 2007** [2]	Wrong intervention, one group in supine and the other only to 40 degrees elevation
**Abruzzi 2017** [3]	Wrong patient population, not severe brain injury
**Adeolu 2012** [4]	Wrong patient population, not severe subdural haematoma
**Agbeko 2012** [5]	Wrong study design, observational study on 10 children
**Allison 2007** [6]	Wrong comparator, both groups mobilised head-up
**Ancona 2019** [7]	Wrong intervention, does not emphasize early mobilisation
**Andelic 2012** [8]	Wrong study design (quasi-randomised), no adverse events reported
**Anderson 2017** [9–15]	Wrong intervention, did not mobilise above 30 degrees
**Asberg 1989** [16]	Wrong study design (quasi-randomised), no adverse events reported
**AVERT-DOSE** [17]	Ongoing trial. Wrong patient population, not severe brain injury
**Awad 2016** [18]	Wrong study design, review
**Bai 2012** [19]	Wrong intervention, does not emphasize early mobilisation
**Bernhardt 2008**[20]	Wrong study design, comparison between stroke units in Trondheim, Norway and Melbourne, Australia
**Bernhardt 2011** [21]	Wrong study design, response to letter
**Bernhardt 2017a** [22]	Wrong study design, response to letter
**Bernhardt 2017b** [23]	Wrong study design, review
**Bernhardt 2017c** [24]	Wrong study design, review
**Borg 2011** [25]	Wrong study design, not patient data
**Brummel 2012** [26]	Wrong study design, study protocol
**Brummel 2014** [27]	Wrong patient population, not severe brain injury
**Chang 2017** [28]	Wrong intervention, not emphasizing early mobilisation
**Chen 2008** [29]	Wrong study design, describes the clinicians experience of participating in a multicentre trial
**Collier 2007** [30]	Wrong study design, no adverse events reported
**Collier 2010a** [31]	Wrong study design, not patient data
**Collier 2010b** [32]	Wrong study design, not patient data
**Collier 2010c** [33]	Wrong study design, not patient data
**Craig 2010** [34]	Wrong study design, systematic review, not unique data
**Cuthbertson 2017** [35]	Wrong study design, editorial
**English 2016** [36]	Wrong patient population, not severe stroke
**Fink 2018** [37]	Wrong intervention, not emphasizing early mobilisation
**Garrote 2016** [38]	Wrong study design, review
**Kumaran 2013** [39]	Wrong patient population, not severe stroke
**Kutlubaev 2015** [40]	Wrong study design, review
**Langhorne 2010** [41]	Wrong patient population, not severe stroke
**Langhorne 2011** [42]	Wrong study design, letter
**Lavados 2014** [43]	Wrong study design, systematic review
**Liang 2005** [44]	Wrong study design, not randomised, no adverse events reported
**Liu 2014** [45]	Wrong patient population, not severe stroke
**Logan 2017** [46]	Wrong comparator, control group received soft mobilisation to sitting position
**Luk'ianov 2010** [47]	Wrong comparator, both groups mobilised head-up
**Martinsson 2003** [48]	Wrong comparator, both groups mobilised head-up
**Mayor 2017** [49]	Wrong study design, review
**Melchers 1999** [50]	Wrong intervention, no early mobilisation as part of the stimulation process

**Meng 2005** [51]	Wrong comparator, both groups mobilised head-up
**Morreale 2016** [52]	Wrong comparator, does not emphasize early mobilisation
**Na 2018** [53]	Wrong intervention, not emphasizing early mobilisation
**Olkowski 2013** [54]	Wrong study design, observational
**Pang 2003** [55]	Wrong intervention, not emphasizing early mobilisation
**Poletto 2016** [56]	Wrong patient population, not severe stroke
**Qi 2012** [57]	Wrong intervention, not specified
**Rocca 2016** [58]	Wrong intervention, does not emphasize earlier mobilisation
**Rybalko 2009** [59]	Wrong comparator, standard care group where mobilised
**Sarfati 2017** [60]	Wrong patient population, not severe brain injury
**Schmidt 2016** [61]	Wrong study design, review
**Seeto 2013** [62]	Wrong study design, observational
**Seo 2006** [63]	Wrong patient population, not severe stroke
**SEVEL 2016** [64]	Wrong patient population, not severe stroke
**Sundseth 2012** [65]	Wrong patient population, not severe stroke
**Thompson 2013** [66]	Wrong study design, observational
**Tong 2017** [67]	Wrong patient population, not severe stroke
**Trevena-Peters 2017** [68]	Wrong intervention, not emphasizing early mobilisation
**Van Vuuren 2016** [69]	Wrong study design, observational
**Venturelli 2015** [70]	Wrong study design, international survey among physicians
**Wang 2004** [71]	Wrong intervention, not emphasizing early mobilisation
**Wang 2005** [72]	Wrong intervention, both groups could potentially do exercises. The control group was asked to exercise
**Wang 2014** [73]	Wrong patient population, not severe stroke
**Wang 2017** [74]	Wrong study design, review
**Witcher 2015** [75]	Wrong study design, observational
**Yelnik 2017** [76]	Wrong comparator, control group received soft mobilisation to sitting position
**Zeng 2007** [77]	Wrong patient population, not severe stroke
**Zhang 2005** [78]	Wrong patient population
**Zhao 2003** [79]	Wrong intervention, no description of the intervention
**Zhong 2006** [80]	Wrong comparator, both groups mobilised head-up
**Zhu 2004** [81]	Wrong comparator, standard care group where mobilised

References of excluded studies

1. Abdulwahab S. Physical disability in patients with hemiparesis. Int J Rehabil Res. 1996;19: 157–161. Available: http://search.ebscohost.com/login.aspx?direct=true&db=cin20&AN=107305169&site=ehost-live NS -
2. Abouzari M, Rashidi A, Rezaii J, Esfandiari K, Asadollahi M, Aleali H, et al. The role of postoperative patient posture in the recurrence of traumatic chronic subdural hematoma after burr-hole surgery. Neurosurgery. 2007;61: 794–7; discussion 797. doi:10.1227/01.NEU.0000298908.94129.67
3. Abruzzi F, J APP, P MF, J SDA, K MB, M SB, et al. Ultra early mobilization reduces the time of mechanical ventilation and ICU stay. Crit Care. 2017;Conference. doi:10.1186/s13054-017-1630-4
4. Adeolu A, Rabiu T, Adeleye A. Post-operative day two versus day seven mobilization after burr-hole drainage of subacute and chronic subdural haematoma in Nigerians. Br J Neurosurg. 2012;26: 743–746. doi:10.3109/02688697.2012.690912
5. Agbeko R, Pearson S, Peters M, McNames J, Goldstein B. Intracranial pressure and cerebral perfusion pressure responses to head elevation changes in pediatric traumatic brain injury. Pediatr Crit care Med. 2012;13: e39-47. doi:10.1097/PCC.0b013e31820ac2ad
6. Allison R, Dennett R. Pilot randomized controlled trial to assess the impact of additional supported standing practice on functional ability post stroke. Clin Rehabil. 2007;21: 614–619. doi:10.1177/0269215507077364

7. Ancona E, Quarenghi A, Simonini M, Saggini R, Mazzoleni S, De Tanti A, et al. Effect of verticalization with Erigo® in the acute rehabilitation of severe acquired brain injury. Neurol Sci. 2019. doi:10.1007/s10072-019-03917-0
8. Andelic N, Bautz-Holter E, Ronning P, Olafsen K, Sigurdardottir S, Schanke AK, et al. Does an early onset and continuous chain of rehabilitation improve the long-term functional outcome of patients with severe traumatic brain injury? J Neurotrauma. 2012;29: 66–74. doi:10.1089/neu.2011.1811
9. Anderson CS, Arima H, Lavados P, Billot L, Hackett ML, Olavarria V V., et al. Cluster-randomized, crossover trial of head positioning in acute stroke. N Engl J Med. 2017;376: 2437–2447. doi:10.1056/NEJMoa1615715
10. Arima H, Lavados P, Middleton S, Watkins C, Robinson T, Heritier S, et al. Rationale of the head position in acute stroke trial (HeadPoST). Cerebrovasc Dis. 2013;36: 31–32. doi:http://dx.doi.org/10.1159/000353795
11. Olavarría V V, Arima H, Anderson CS, Brunser A, Muñoz-Venturelli P, Billot L, et al. Statistical analysis plan of the head position in acute ischemic stroke trial pilot (HEADPOST pilot). Int J Stroke. 2017;12: 211–215. doi:10.1177/1747493016674955
12. Brunser AM, Muñoz Venturelli P, Lavados PM, Gaete J, Martins S, Arima H, et al. Head position and cerebral blood flow in acute ischemic stroke patients: Protocol for the pilot phase, cluster randomized, Head Position in Acute Ischemic Stroke Trial (HeadPoST pilot). Int J Stroke. 2016;11: 253–259. doi:10.1177/1747493015620808
13. Munoz Venturelli P, Arima H, Lim J, Lavados P, Middleton S, Watkins C, et al. The Head Position in Acute Stroke Trial (HeadPOST): background and rationale. Int J stroke. 2014;9 Suppl 1: 38. doi:10.1111/ijs.12298
14. Munoz-Venturelli P, Arima H, Lavados P, Brunser A, Peng B, Cui L, et al. Head Position in Stroke Trial (HeadPoST)--sitting-up vs lying-flat positioning of patients with acute stroke: study protocol for a cluster randomised controlled trial. Trials [electronic Resour. 2015;16: 256 CNO-CN. doi:10.1186/s13063-015-0767-1
15. Olavarria V, PM L, PM M-V, Gaete S, Martins S, Arima H, et al. Head position in stroke trial (HEADPOST) pilot phase. Int J stroke. 2015;10: 430. doi:10.1111/ijs.12479
16. KH A. Orthostatic tolerance training of stroke patients in general medical wards. An experimental study. Scand J Rehabil Med. 1989;21: 179–185. Available: NS -
17. ANZCTR. A Phase 3, Multi Arm, Multi Stage, Covariate Adjusted, Response Adaptive, Randomised Trial to Determine Optimal Early Mobility Training after Stroke. In: Australian New Zealand Clinical Trials Registry [Internet]. [cited 28 Feb 2020]. Available: https://www.anzctr.org.au/TrialSearch.aspx#&&searchTxt=AVERT+DOSE&registry=&interventionDescription=&interventionCodeOperator=OR&interventionCode=&studyType=&allocationToIntervention=&recruitmentStatus=&healthCondition=&conditionCategory=&conditionCode=&g
18. Awad A, Kellner C, Mascitelli J, Bederson J, Mocco J. No early mobilization after stroke: lessons learned from the AVERT trial. World Neurosurg. 2016;87: 474. Available: NS -
19. Bai Y, Hu Y, Wu Y, Zhu Y, He Q, Jiang C, et al. A prospective, randomized, single-blinded trial on the effect of early rehabilitation on daily activities and motor function of patients with hemorrhagic stroke. J Clin Neurosci. 2012;19: 1376–1379. doi:10.1016/j.jocn.2011.10.021
20. Bernhardt J, Chitravas N, IL M, AG T, Indredavik B. Not all stroke units are the same - A comparison of physical activity patterns in Melbourne, Australia, and Trondheim, Norway. Stroke. 2008; 2059–2065. doi:10.1161/STROKEAHA.107.507160
21. Bernhardt J, Cumming T, Ha J. Response to Letter by Freeman et al Regarding Article, 'Very Early Mobilization After Stroke Fast-Tracks Return to Walking: Further Results From the Phase II AVERT Randomized Controlled Trial'. Stroke. 2011;42: e585. Available: NS -
22. Bernhardt J. Response to letter: And yet it moves – AVERT enlightens translations stroke research. Int J Stroke. 2017;12: NP14-NP15. doi:10.1177/1747493015621453
23. Bernhardt J. Early mobilisation and rehabilitation in intensive care unit-ready for implementation? Ann Transl Med. 2017;5. doi:10.21037/atm.2017.01.48
24. Bernhardt J, Godecke E, Johnson L, Langhorne P. Early rehabilitation after stroke. Curr Opin Neurol. 2017;30: 48–54. doi:10.1097/WCO.0000000000000404
25. Borg J, Roe C, Nordenbo A, Andelic N, C de B, JL af G. Trends and Challenges in the Early Rehabilitation of Patients with Traumatic Brain Injury A Scandinavian Perspective. Am J Phys Med Rehabil. 2011; 65–73. doi:10.1097/PHM.0b013e3181fc80e7
26. Brummel NE, Jackson JC, Girard TD, Pandharipande PP, Schiro E, Work B, et al. A Combined Early Cognitive and Physical Rehabilitation Program for People Who Are Critically Ill: The Activity and Cognitive Therapy in the Intensive Care Unit (ACT-ICU) Trial. Phys Ther. 2012;92: 1580–1592. doi:10.2522/ptj.20110414
27. Brummel NE, Girard TD, Ely EW, Pandharipande PP, Morandi A, Hughes CG, et al. Feasibility and safety of early

combined cognitive and physical therapy for critically ill medical and surgical patients: the Activity and Cognitive Therapy in ICU (ACT-ICU) trial. Intensive Care Med. 2014;40: 370–379. doi:10.1007/s00134-013-3136-0

28. Chang S, Zhang W, Fu A. Application of early nursing intervention on patients with cerebral infarction in in training process of language and limb function rehabilitation. Biomed Res. 2017;28: 7523–7525. Available: http://www.biomedres.info/biomedical-research/application-of-early-nursing-intervention-on-patients-with-cerebral-infarction-in-in-training-process-of-language-and-limb-functio.pdf NS -
29. Chen R. Taking the ivory tower of academic research into the clinical world: clinicians' experiences of participating in an international rehabilitation trial (AVERT). Intern Med J. 2008;38: A75. Available: NS -
30. Collier J, Bernhardt J. Does acute stroke unit care change during a rehabilitation clinical trial (AVERT Phase II)? Cerebrovasc Dis. 2007;23: 96. Available: NS -
31. Collier J, Speare S, Churilov L, Bernhardt J. What stops patients being recruited to an early rehabilitation trial?: preliminary results from an ongoing phase III RCT (AVERT). Cerebrovasc Dis. 2010;29: 60. Available: NS -
32. Collier J. The practical challenges of ensuring high quality data for a large multinational stroke rehabilitation trial (AVERT). Int J Stroke. 2010;5: 4. Available: NS -
33. Collier J. Can more stroke patients be recruited into an ongoing trial of very early rehabilitation (AVERT)? Int J Stroke. 2010;5: 31. Available: NS -
34. Craig LE, Bernhardt J, Langhorne P, Wu O. Early Mobilization After Stroke. Stroke. 2010;41: 2632–2636. doi:10.1161/STROKEAHA.110.588244
35. Cuthbertson BH, Goddard S. Benefits and harms of early rehabilitation. Intensive Care Med. 2017;43: 1878–1880. doi:10.1007/s00134-017-4904-z
36. English C, GN H, Olds T, Parfitt G, Borkoles E, Coates A, et al. Reducing sitting time after stroke: a phase II safety and feasibility randomized controlled trial. Arch Phys Med Rehabil. 2016;97: 273–280. doi:10.1016/j.apmr.2015.10.094
37. Fink E, Beers S, Houtrow A, Richichi R, Burns C, Doughty L, et al. Pilot RCT of early versus usual care rehabilitation in pediatric neurocritical care. Crit Care Med. 2018;Conference: 394. doi:10.1097/01.ccm.0000528828.59765.02
38. Divison Garrote J, Escobar Cervantes C. Efficacy and safety of early mobilisation after stroke onset (AVERT): a randomised controlled trial. Semer Soc Esp Med Rural y Gen. 2016;42: 482–484. doi:10.1016/j.semerg.2015.11.016
39. Kumaran P, Vanan M. Effect of early mobilisation training on gross motor function and functional outcome in hemi paretic stroke patients. Int J Pharm Technol. 2013;5: 5637–5650. Available: http://onlinelibrary.wiley.com/o/cochrane/clcentral/articles/969/CN-00979969/frame.html NS -
40. Kutlubaev MA, Akhmadeeva LR. The early post-stroke mobilization. Vopr Kurortol Fizioter i Lech Fiz kul'tury. 2015;92: 46. doi:10.17116/kurort2015146-50
41. Langhorne P, Stott D, Knight A, Bernhardt J, Barer D, Watkins C. Very early rehabilitation or intensive telemetry after stroke: a pilot randomised trial. Cerebrovasc Dis. 2010;29: 352–360. doi:10.1159/000278931
42. Langhorne P, Stott D. Letter by Langhorne and stott regarding article, 'very early mobilization after stroke fast-tracks return to walking: Further results from the phase ii avert randomized controlled trial'. Stroke. 2011;42: e376. doi:http://dx.doi.org/10.1161/STROKEAHA.111.613554
43. Lavados P, VV O, Arima H, Brunser A, Munoz-Venturelli P, Heritier S, et al. Head position and cerebral blood flow velocity in acute ischemic stroke: a systematic review and meta-analysis. Cerebrovasc Dis Conf 23th Eur stroke Conf Fr Conf start 20140506 Conf end 20140509. 2014;Conference: 262–263. Available: NS -
44. Liang S, Peng X, Yang J. Two-stage rehabilitative training in the recovery of walking ability in stroke patients with hemiplegia. Chinese J Clin Rehabil. 2005;9: 8–9. Available: NS -
45. Liu N, DA C, NE A, Zeng L, Li Z, Li J, et al. Randomized controlled trial of early rehabilitation after intracerebral hemorrhage stroke: difference in outcomes within 6 months of stroke. Stroke. 2014;45: 3502–3507. doi:10.1161/STROKEAHA.114.005661
46. Logan A, Freeman J, Kent B, Pooler J, Creaner S, Vickery J, et al. A randomised controlled feasibility trial to investigate the effects of a functional standing frame programme versus usual physiotherapy in people with severe sub-acute stroke. 2017; 88–89. Available: NS -
47. Luk'ianov A, Shamalov N, Ivanova G, Skvortsova V. Passive tilting in patients in the acute period of cerebral stroke. Zh Nevrol Psikhiatr Im S S Korsakova. 2010;S. Korsako: 29–35. Available: NS -
48. Martinsson L, Eksborg S, NG W. Intensive early physiotherapy combined with dexamphetamine treatment in severe stroke: a randomized, controlled pilot study. Cerebrovasc Dis. 2003;16: 338–345. doi:72555

49. Mayor S. Lying flat after stroke achieves similar outcomes to sitting up, trial finds. BMJ. 2017;357. doi:10.1136/bmj.j3051
50. Melchers P, Maluck A, Suhr L, Scholten S, Lehmkuhl G. An early onset rehabilitation program for children and adolescents after traumatic brain injury (TBI): Methods and first results. Restor Neurol Neurosci. 1999;14: 153–160. Available: NS -
51. Meng Z, Ni C, Li J, Han R. Effects of early rehabilitation on the functional outcomes of stroke patients estimated with functional comprehensive assessment. [Chinese]. Chinese J Clin Rehabil. 2005;9: 1–3. Available: NS -
52. Morreale M, Marchione P, Pili A, Lauta A, Castiglia SF, Spallone A, et al. Early versus delayed rehabilitation treatment in hemiplegic patients with ischemic stroke: proprioceptive or cognitive approach? Eur J Phys Rehabil Med. 2016;52: 81–9. Available: http://www.ncbi.nlm.nih.gov/pubmed/26220327
53. Na K, He J, Hu L, Wu L, Li Y, Zhao D, et al. Early treatment of acute ischemic stroke by integrated traditional and western medicine. Int J Clin Exp Med. 2018;11: 2901–2907. Available: NS -
54. Olkowski BF, Devine MA, Slotnick LE, Veznedaroglu E, Liebman KM, Arcaro ML, et al. Safety and Feasibility of an Early Mobilization Program for Patients With Aneurysmal Subarachnoid Hemorrhage. Phys Ther. 2013;93: 208–215. doi:10.2522/ptj.20110334
55. Pang G. Early rehabilitation care effect on ability of daily life of stroke patients. Chinese J Clin Rehabil. 2003;7: 1230–1231. Available: NS -
56. Poletto SR, Rebello LC, Valenca MJM, Rossato D, Almeida AG, Brondani R, et al. Early Mobilization in Ischemic Stroke: A Pilot Randomized Trial of Safety and Feasibility in a Public Hospital in Brazil. Cerebrovasc Dis Extra. 2015;5: 31–40. doi:10.1159/000381417
57. Qi G. Early phase rehabilitation improves limb motor function and self-care capability for patients with severe traumatic brain injury. Brain Inj. 2012;26: 405. doi:10.3109/02699052.2012.660091
58. Rocca A, JM P, Berney L, Johr J, D V de V, RT D, et al. Sympathetic activity and early mobilization in patients in intensive and intermediate care with severe brain injuries: a preliminary prospective randomized study. BMC Neurol. 2016;16: 169. doi:10.1186/s12883-016-0684-2
59. Rybalko N V, Daminov VD, Kuznetsov AN. Estimation of safety and efficacy application tilt-table Erigo in acute stroke. J Neurol Sci. 2009;285: S179–S180. Available: NS -
60. Sarfati C, Moore A, Mendialdua P, Rodet E, Pilorge C, Stephan F, et al. Study of efcacy on ICU acquired weakness of early standing with the assistance of a tilt table in critically ill patients. Ann intensive care Conf french intensive care Soc Int Congr - Reanim 2017 Fr Conf start 20170111 Conf end 20170113. 2017;Conference: 206–207. doi:10.1186/s13613-016-0224-7
61. Schmidt A, Minnerup J. And yet it moves--AVERT enlightens translational stroke research. Int J stroke. 2016;11: np39. doi:10.1177/1747493015621067
62. Treena S, Suzanne K, Cassandra B, Erin G, Haylee K, Steven M. Feasibility of an interdisciplinary early intervention for patients with low levels of responsiveness following an acquired brain injury. Brain Impair. 2013;14: 213–221. doi:10.1017/BrImp.2013.20
63. Seo N, Han M, Lee J. Effects of a tilting training program on lower extremities function, depression, and self-efficacy among stroke inpatients. Taehan Kanho Hakhoe Chi. 2006;36: 514–522. Available: NS -
64. Herisson F, Godard S, Volteau C, Le Blanc E, Guillon B, Gaudron M, et al. Early Sitting in Ischemic Stroke Patients (SEVEL): A Randomized Controlled Trial. PLoS One. 2016;11: e0149466. doi:10.1371/journal.pone.0149466
65. Sundseth A, Thommessen B, OM R. Mobilisation within 24 hours of acute stroke. A randomised controlled trial. Akershus mobilisation in stroke study (AKEMIS). Cerebrovasc Dis. 2012;33: 623–624. doi:10.1159/000339538
66. Thompson JN, Majumdar J, Sheldrick R, Morcos F. Acute neurorehabilitation versus treatment as usual. Br J Neurosurg. 2013;27: 24–29. doi:10.3109/02688697.2012.714818
67. Tong Y, JY D, MG S, Du H, Geng X, Ding Y. Early but not too early, high intensity physical exercise rehabilitation may be beneficial: a pilot clinical study. Stroke. 2017;Conference.
68. Trevena-Peters J, Ponsford J, McKay A. Agitated Behavior and Activities of Daily Living Retraining During Posttraumatic Amnesia. J Head Trauma Rehabil. 2017. doi:10.1097/htr.0000000000000363
69. Van Vuuren J, Santegoets K, De Laat F. The outcome of level of consciousness after early intensive neurorehabilitation programme (EINP). Brain Inj Conf 11th world Congr brain Inj Int brain Inj Assoc Netherlands Conf start 20160302 Conf end 20160305. 2016;Conference: 542. doi:10.3109/02699052.2016.1162060

70. Muñoz Venturelli P, Olavarría V, González F, Brunser A, Lavados P, Arima H, et al. Head Position in the Early Phase of Acute Ischemic Stroke: An International Survey of Current Practice. J Stroke Cerebrovasc Dis. 2015;24: 1564–1569. doi:10.1016/j.jstrokecerebrovasdis.2015.03.023
71. Wang Q, Gan Z, Lu H, Hu X, Liu H, Cai C, et al. Effect of early exercise therapy on the recovery of motor function in patients with cerebral infarction and the changes of somatosensory evoked potential. Chinese J Clin Rehabil. 2004;8: 6023–6025. Available: NS -
72. Wang B, Li H, Xu B, H-P S, Cao Y, Xu Y. Influence of earlier rehabilitative interventions on the emotions and the ability of daily living of patients following the first onset of acute stroke. Chinese J Clin Rehabil. 2005;9: 176–178. Available: NS -
73. Wang Z, Wang L, Fan H, Jiang W, Wang S, Gu Z, et al. Adapted Low Intensity Ergometer Aerobic Training for Early and Severely Impaired Stroke Survivors: A Pilot Randomized Controlled Trial to Explore Its Feasibility and Efficacy. J Phys Ther Sci. 2014;26: 1449–1454. doi:10.1589/jpts.26.1449
74. Wang W, Wu J. Study on early rehabilitation and rehabilitation technology after stroke: chinese scholars' reports published abroad. Chinese J Contemp Neurol Neurosurg. 2017;17: 166–170. doi:10.3969/j.issn.1672-6731.2017.03.002
75. Witcher R, Stoerger L, AL D, Silverstein A, Rosengart A, Brodie D, et al. Effect of early mobilization on sedation practices in the neurosciences intensive care unit: a preimplementation and postimplementation evaluation. J Crit Care. 2015; 344–347. doi:10.1016/j.jcrc.2014.12.003
76. Yelnik AP, Quintaine V, Andriantsifanetra C, Wannepain M, Reiner P, Marnef H, et al. AMOBES (Active Mobility Very Early After Stroke). Stroke. 2017;48: 400–405. doi:10.1161/STROKEAHA.116.014803
77. Zeng X. The effect of early mobilization for stroke patients. 2007. Available: http://www.chictr.org.cn/showprojen.aspx?proj=9324 NS -
78. Zhang D, Zhu S, Cui G, Liu S, Li Y. Difference between early and late rehabilitative intervention in ameliorating the motor function and activities of daily living in patients with cerebral infarction. Chinese J Clin Rehabil. 2005;9: 149–151. Available: NS -
79. Zhao F, Wang L, Tian G, Zhou J, Han J. Early rehabilitation intervention promoting ability of daily living in acute stroke patients. Chinese J Clin Rehabil. 2003;7: 851. Available: NS -
80. Zhong M, Mo H, Liu H. Evidence-based nursing on early rehabilitation of cerebral infarction patients with hemiplegia. Chinese Nurs Res. 2006;20: 393–395. Available: http://search.ebscohost.com/login.aspx?direct=true&db=cin20&AN=106216132&site=ehost-live NS -
81. Zhu G, Hu Y, Wu Y, Zhu Y, Han X, Sun L, et al. Effects of standardized three-stage rehabilitation on recovery of neurological function in stroke patients with hemiplegia. Natl Med J China (zhonghua Yi Xue Za Zhi). 2004;84: 1955–1958. Available: http://onlinelibrary.wiley.com/o/cochrane/clcentral/articles/869/CN-00527869/frame.html NS -

Fig 1 to 13. Trial Sequential Analysis.

Fig 1. Trial Sequential Analysis. Comparison of early mobilisation versus standard care – Mortality or poor functional outcome at the end of intervention

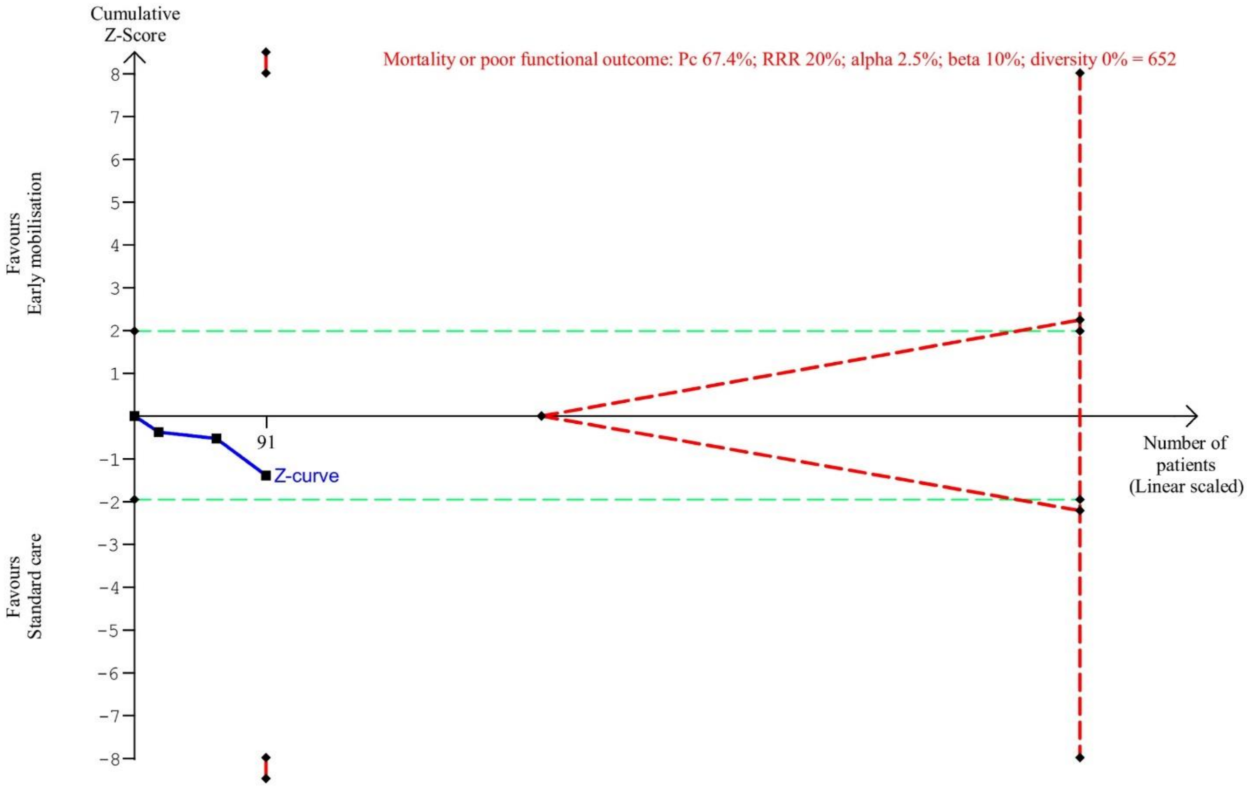

Trial Sequential Analysis of the cumulative fixed-effect meta-analysis assessing early mobilisation versus standard care on mortality or poor functional outcome in patients with severe acquired brain injury is based on a proportion in the control group (Pc) of 67.4%, a relative risk reduction (RRR) of 20%, an alpha of 2.5%, a beta of 10%, and diversity of 0% and results in a required information size of 652 patients. The cumulative Z-curve (blue) does not pass the boundaries for benefit (upper red line), harm (lower red line), or futility (the two outward sloping dashed red lines at the right).

Fig 2. Comparison of early mobilisation versus standard care – Mortality or poor functional outcome at maximal follow-up

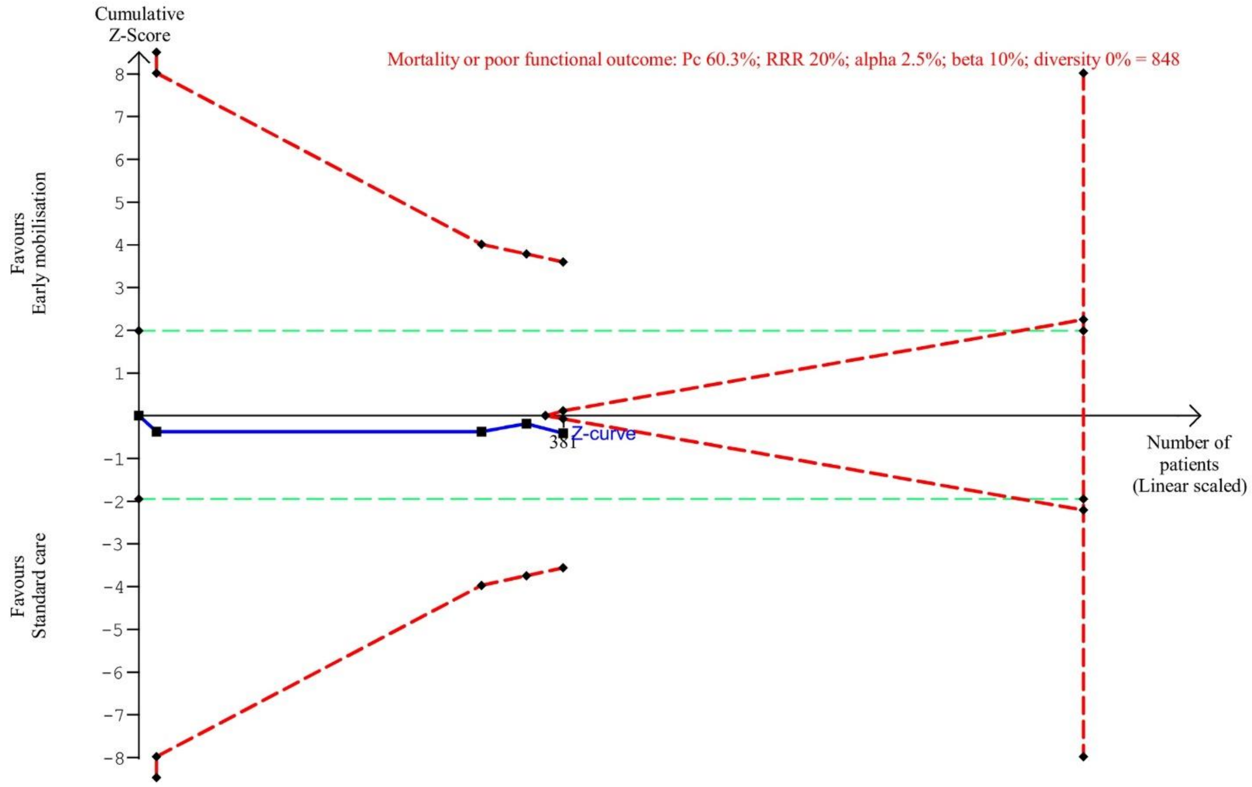

Trial Sequential Analysis of the cumulative fixed-effect meta-analysis assessing early mobilisation versus standard care on mortality or poor functional outcome in patients with severe acquired brain injury is based on a proportion in the control group (Pc) of 60.3%, a relative risk reduction (RRR) of 20%, an alpha of 2.5%, a beta of 10%, and diversity of 0% and results in a required information size of 848 patients. The cumulative Z-curve (blue) is close to entering the area of futility (the two outward sloping dashed red lines at the right) but is far from the barriers of benefit (upper red line) or harm (lower red line).

Fig 3. Comparison of early mobilisation versus standard care – Mortality at the end of the intervention.

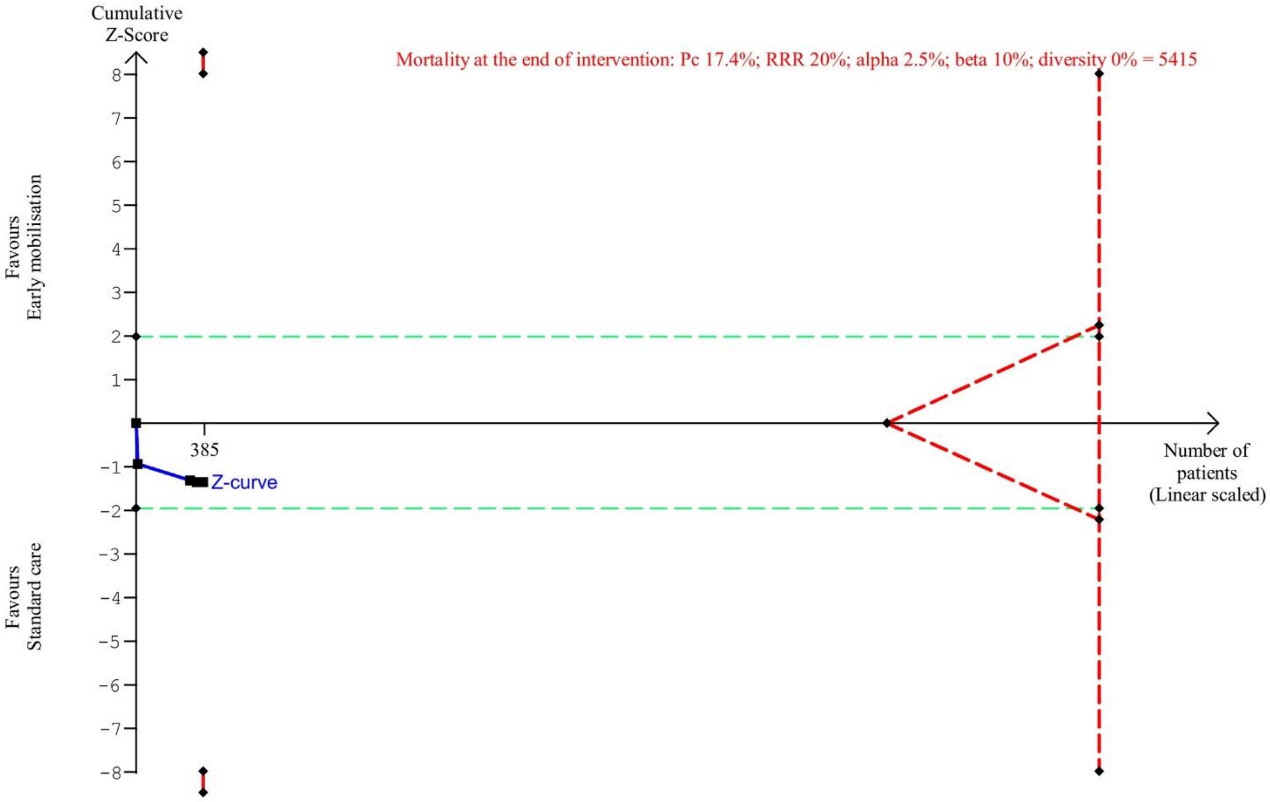

Trial Sequential Analysis of the cumulative fixed effect meta-analysis assessing early mobilisation versus standard care on mortality in patients with severe acquired brain injury is based on a proportion in the control group (Pc) of 17.4%, a relative risk reduction (RRR) of 20%, an alpha of 2.5%, a beta of 10%, and diversity of 0% and results in a required information size of 5415 patients; The cumulative Z-curve (blue) is far from breaking any boundaries for benefit (upper red line), harm (lower red line), or futility (the two outward sloping dashed red lines at the right).

Fig 4. Comparison of early mobilisation versus standard care – Mortality at maximal follow-up.

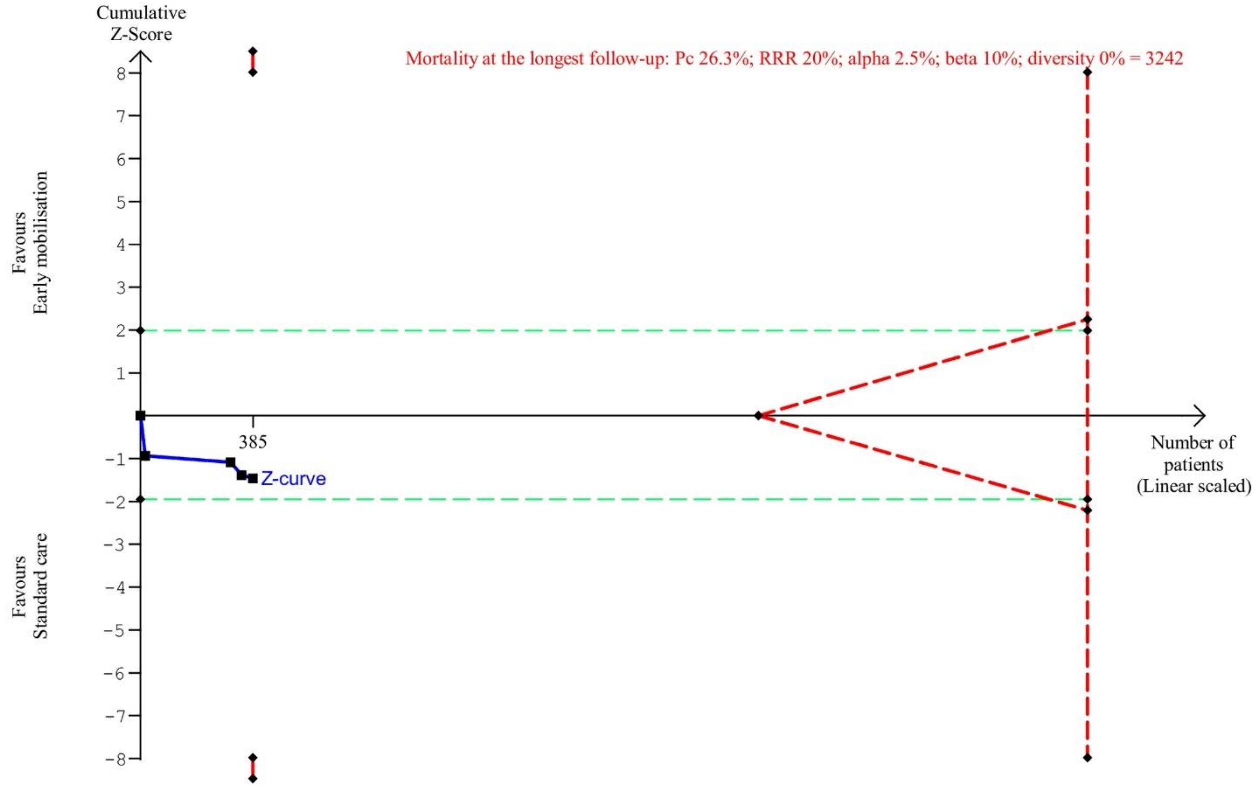

Trial Sequential Analysis of the cumulative fixed-effect meta-analysis assessing early mobilisation versus standard care on mortality in patients with severe acquired brain injury. The analysis is based on a proportion in the control group (Pc) of 26.3%, a relative risk reduction (RRR) of 20%, an alpha of 2.5%, a beta of 10%, and diversity of 0% and results in a required information size of 3242 patients. The cumulative Z-curve (blue) is far from breaking any boundaries for benefit (upper red line), harm (lower red line), or futility (the two outward sloping dashed red lines at the right).

Fig 5. Comparison of early mobilisation versus standard care – Poor functional outcome among survivors at the end of the intervention.

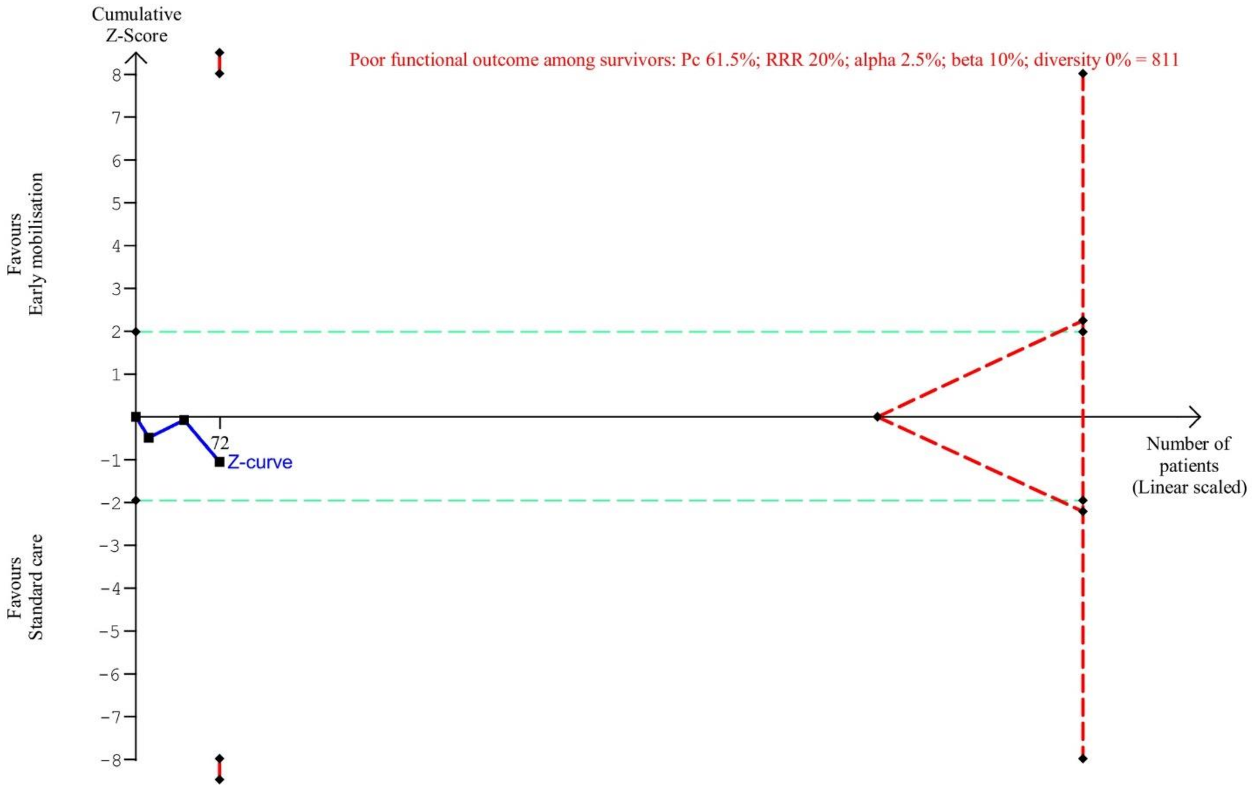

Trial Sequential Analysis of the cumulative fixed-effect meta-analysis assessing early mobilisation versus standard care on poor functional outcome in patients with severe acquired brain injury is based on a proportion in the control group (Pc) of 61.5%, a relative risk reduction (RRR) of 20%, an alpha of 2.5%, a beta of 10%, and diversity of 0% and results in a required information size of 811 patients. The cumulative Z-curve (blue) is far from breaking any boundaries for benefit (upper red line), harm (lower red line), or futility (the two outward sloping dashed red lines at the right).

Fig 6. Comparison of early mobilisation versus standard care – Poor functional outcome among survivors at maximal follow-up.

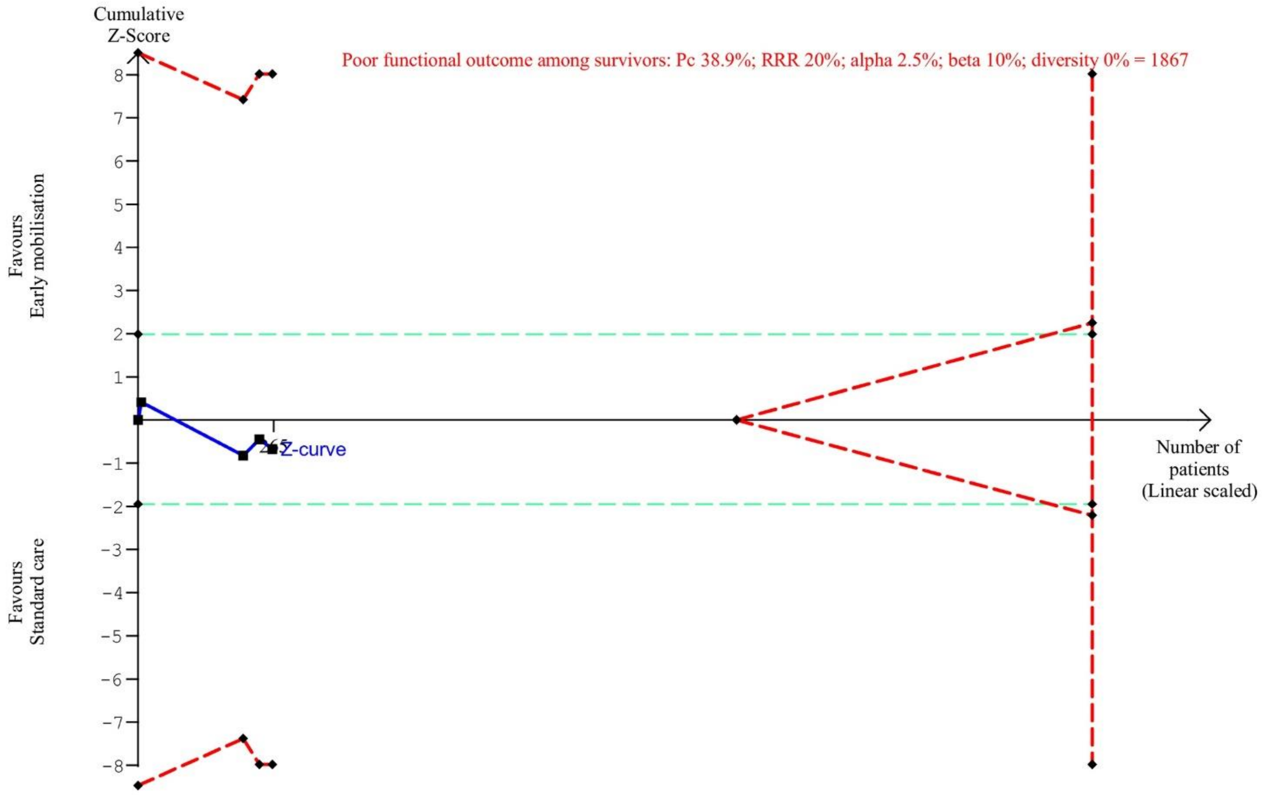

Trial Sequential Analysis of the cumulative fixed-effect meta-analysis assessing early mobilisation versus standard care on poor functional outcome among survivors in patients with severe acquired brain injury is based on a proportion in the control group (Pc) of 38.9%, a relative risk reduction (RRR) of 20%, an alpha of 2.5%, a beta of 10%, and diversity of 0% and results in a required information size of 1867 patients. The cumulative Z-curve (blue) is far from breaking any boundaries for benefit (upper red line), harm (lower red line), or futility (the two outward sloping dashed red lines at the right).

Fig 7. Comparison of early mobilisation versus standard care – Quality of life at maximal follow-up.

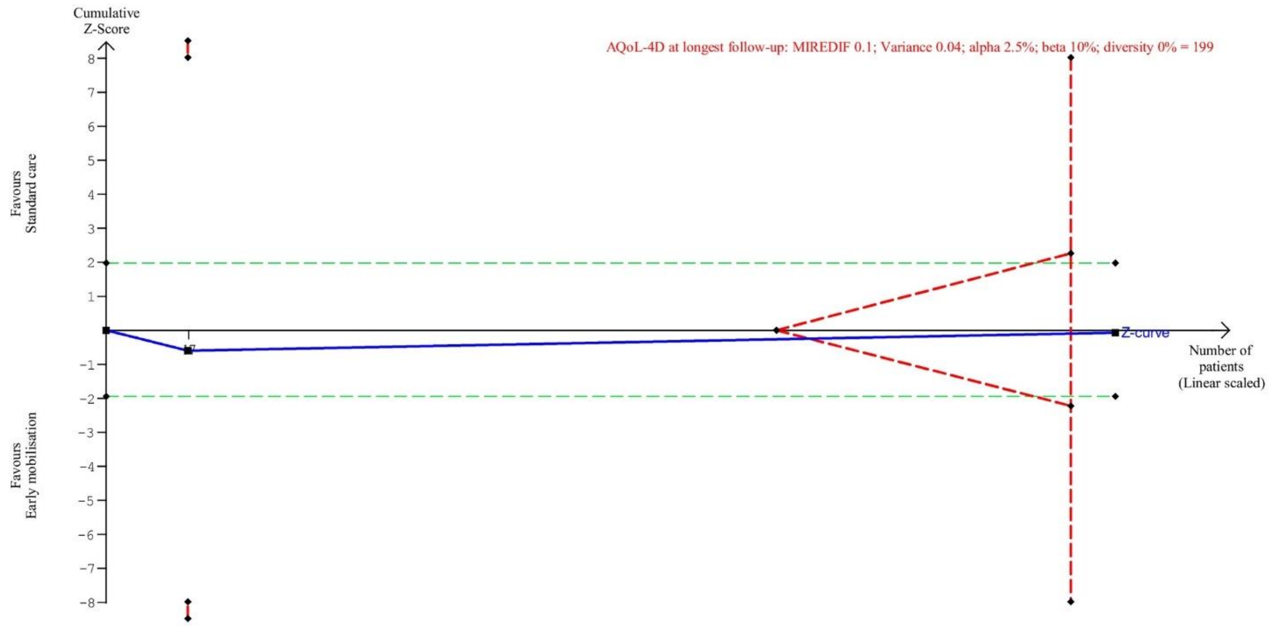

Trial Sequential Analysis of the cumulative fixed-effect meta-analysis assessing early mobilisation versus standard care on quality of life in patients with severe stroke is based on a minimal relevant difference (MIREDIF) of 0.1 and a variance of 0.04, calculated from the control group data, an alpha of 2.5%, a beta of 10%, and diversity of 0% and equals 199 patients. The cumulative Z-curve (blue) enters the area of futility (the two outward sloping dashed red lines at the right) and goes beyond the line of required information size (the red vertical line).

Fig 8. Comparison of early mobilisation versus standard care – Serious adverse events at the end of the intervention.

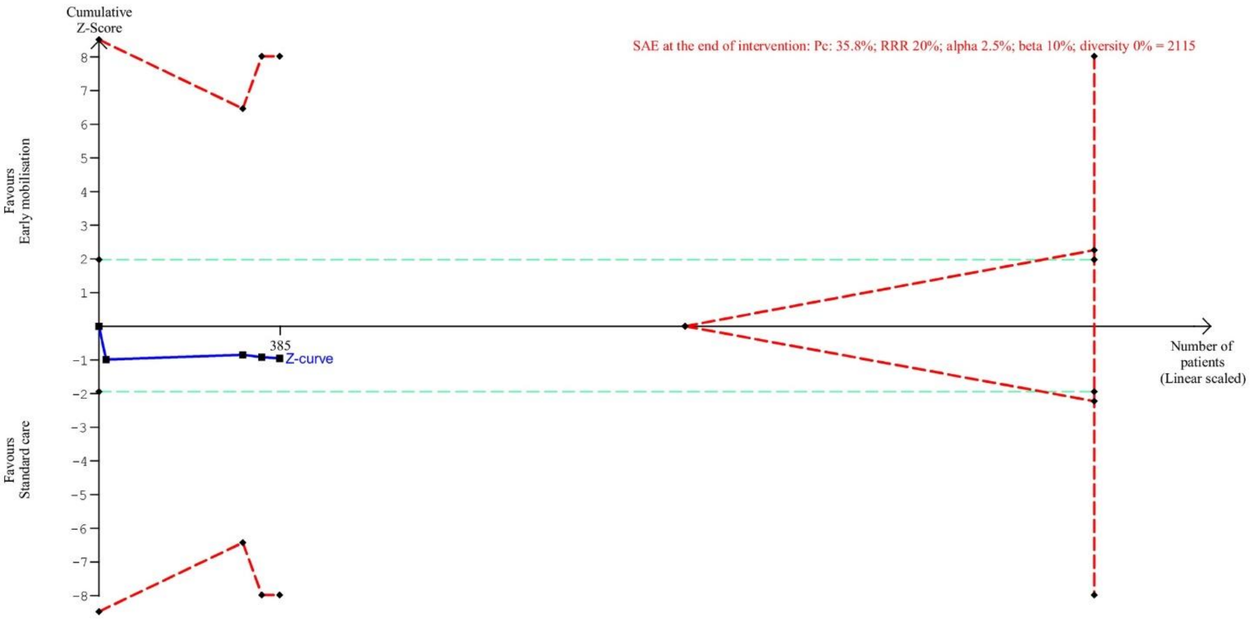

Trial Sequential Analysis (C) of the cumulative fixed-effect meta-analysis assessing early mobilisation versus standard care on serious adverse events in patients with severe acquired brain injury is based on a proportion in the control group (Pc) of 35.8%, a relative risk reduction (RRR) of 20%, an alpha of 2.5%, a beta of 10%, and diversity of 0% and results in a required information size of 2115 patients. The cumulative Z-curve (blue) is far from breaking any boundaries for benefit (upper red line), harm (lower red line), or futility (the two outward sloping dashed red lines at the right).

Fig 9. Comparison of early mobilisation versus standard care – Serious adverse events at maximal follow-up.

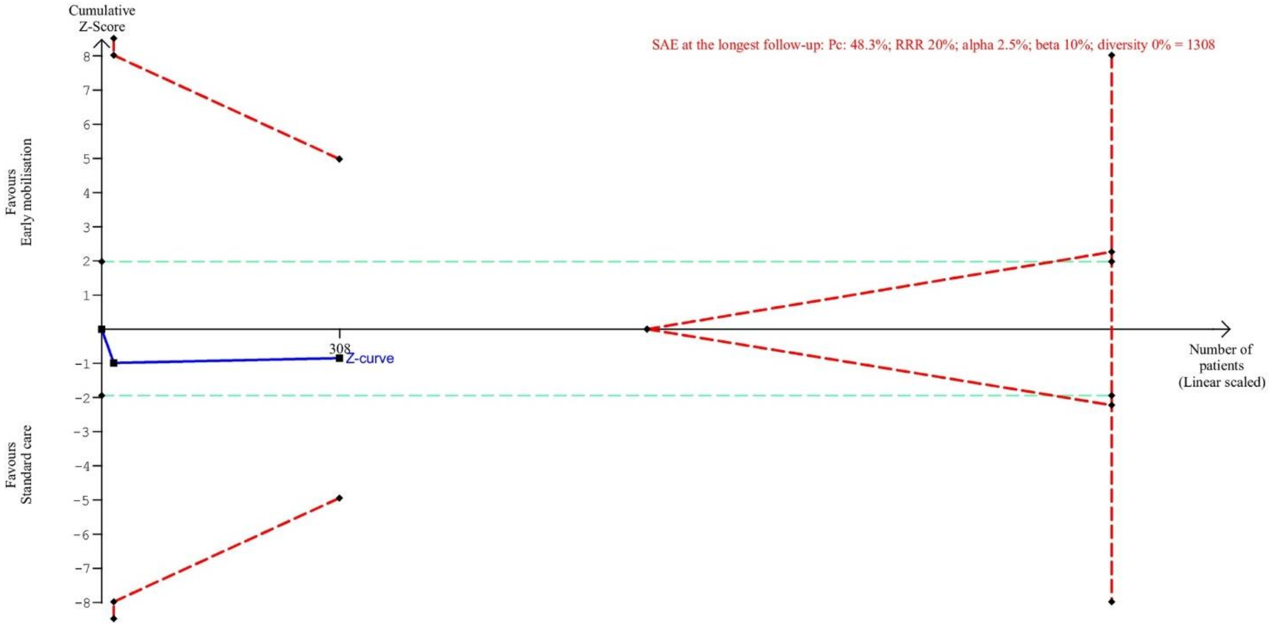

Trial Sequential Analysis of the cumulative fixed-effect meta-analysis assessing early mobilisation versus standard care on serious adverse events in patients with stroke is based on a proportion in the control group (Pc) of 48.3%, a relative risk reduction (RRR) of 20%, an alpha of 2.5%, a beta of 10%, and diversity of 0% and results in a required information size of 1308 patients. The cumulative Z-curve (blue) is far from breaking any boundaries for benefit (upper red line), harm (lower red line), or futility (the two outward sloping dashed red lines at the right).

Fig 10. Comparison of early mobilisation versus standard care – Coma Recovery Scale-Revised at the end of the intervention.

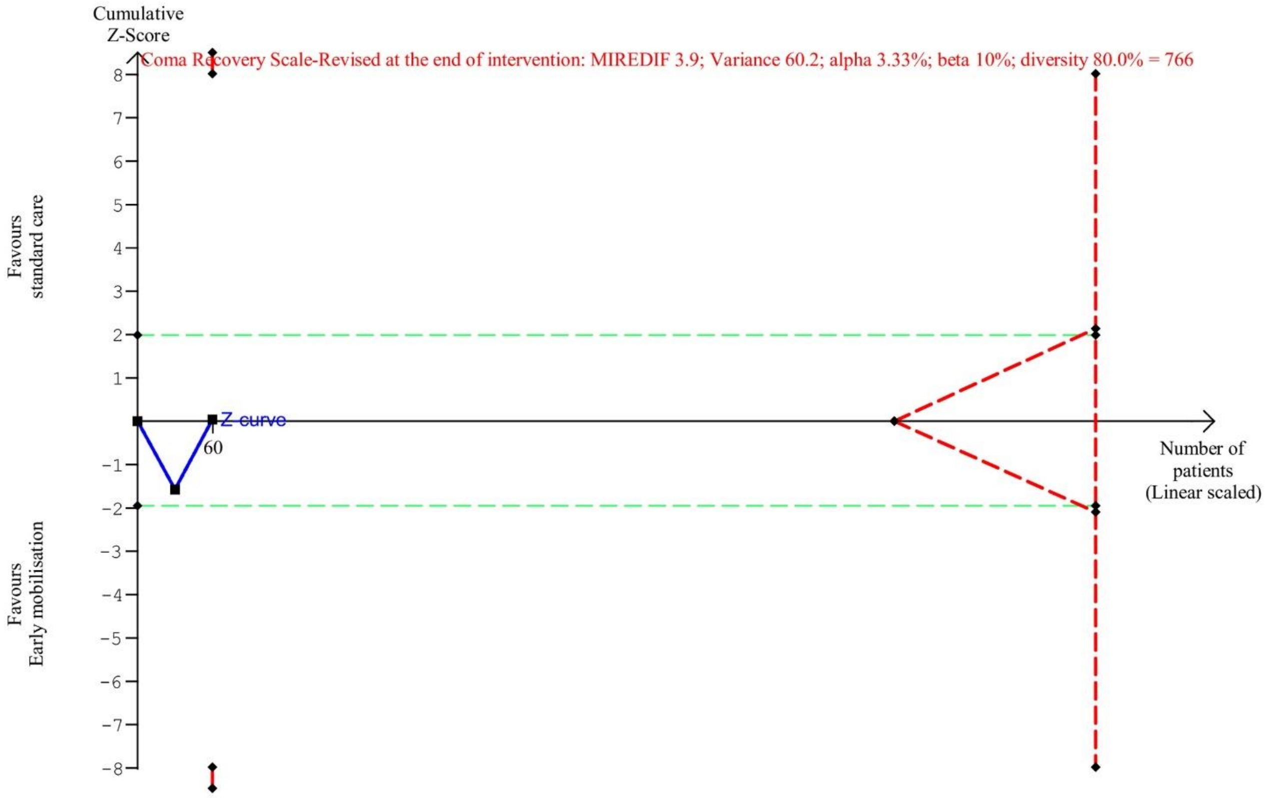

Trial Sequential Analysis of the cumulative random-effects meta-analysis assessing early mobilisation versus standard care on Coma Recovery Scale-Revised in patients with severe acquired brain injury is based on a minimal relevant difference (MIREDIF) of 3.9 and a variance of 60.2, calculated from the control group data, an alpha of 2.5%, a beta of 10%, and diversity of 80% and equals 816 patients. The cumulative Z-curve (blue) is far from breaking any boundaries for benefit (upper red line), harm (lower red line), or futility (the two outward sloping dashed red lines at the right).

Fig 11. Comparison of early mobilisation versus standard care – Coma Recovery Scale-Revised at maximal follow-up.

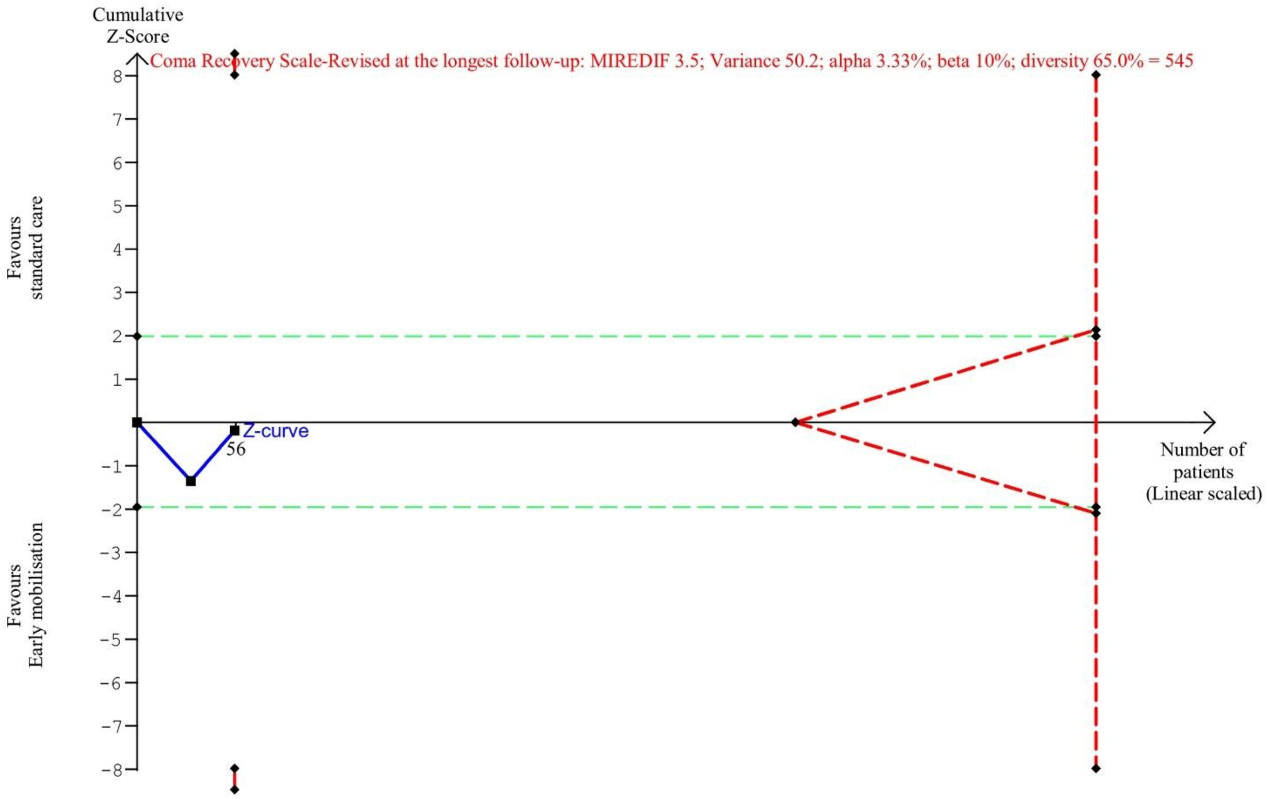

Trial Sequential Analysis of the cumulative random-effects meta-analysis assessing early mobilisation versus standard care on Coma Recovery Scale-Revised in patients with severe acquired brain injury is based on a minimal relevant difference (MIREDIF) of 3.5 and a variance of 50.2, calculated from the control group data, an alpha of 2.5%, a beta of 10%, and diversity of 65% and equals 582 patients. The cumulative Z-curve (blue) is far from breaking any boundaries for benefit (upper red line), harm (lower red line), or futility (the two outward sloping dashed red lines at the right).

Fig 12. Comparison of early mobilisation versus standard care – Adverse events not considered serious at the end of the intervention.

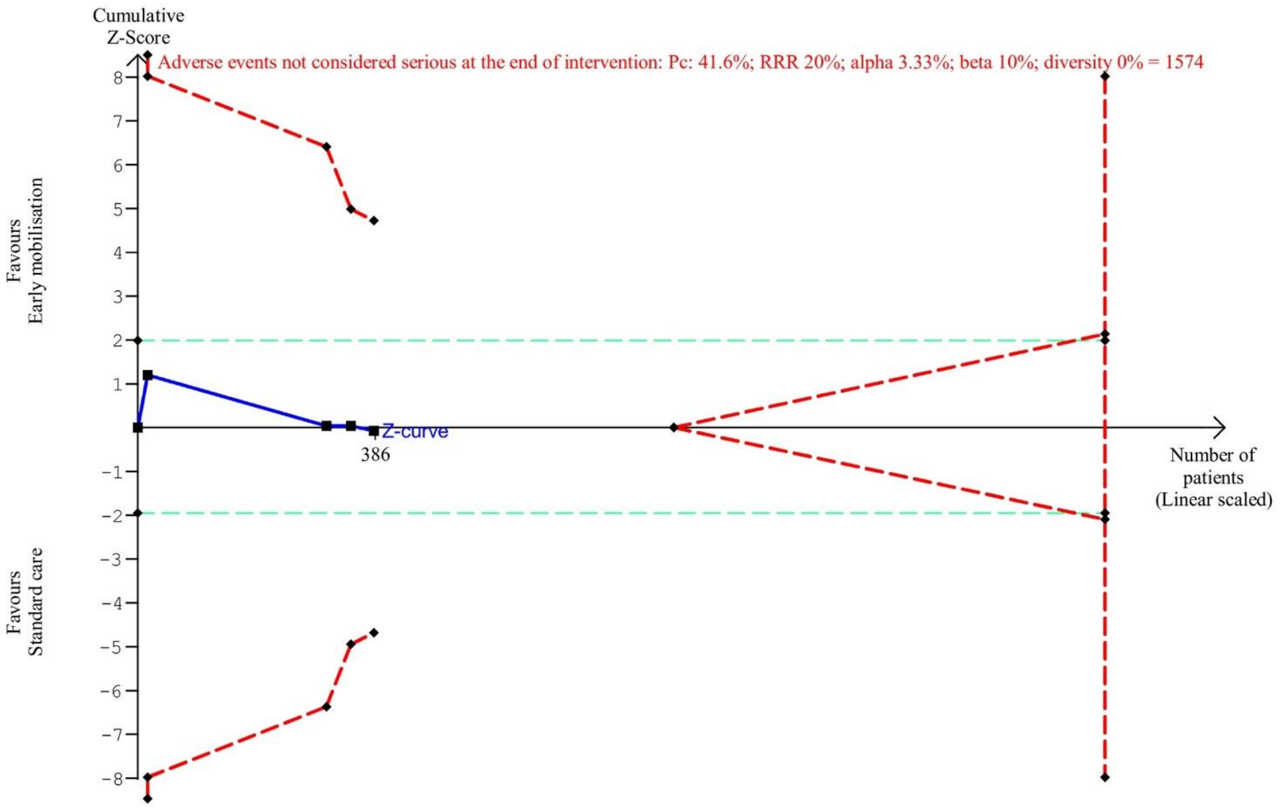

Trial Sequential Analysis of the cumulative fixed-effect meta-analysis assessing early mobilisation versus standard care on adverse events not considered serious in patients with severe acquired brain injury is based on a proportion in the control group (Pc) of 41.6%, a relative risk reduction (RRR) of 20%, an alpha of 2.5%, a beta of 10%, and diversity of 0% and equals 1680 patients. The cumulative Z-curve (blue) is far from breaking any boundaries for benefit (upper red line), harm (lower red line), or futility (the two outward sloping dashed red lines at the right).

Fig 13. Comparison of early mobilisation versus standard care – Adverse events not considered serious at maximal follow-up.

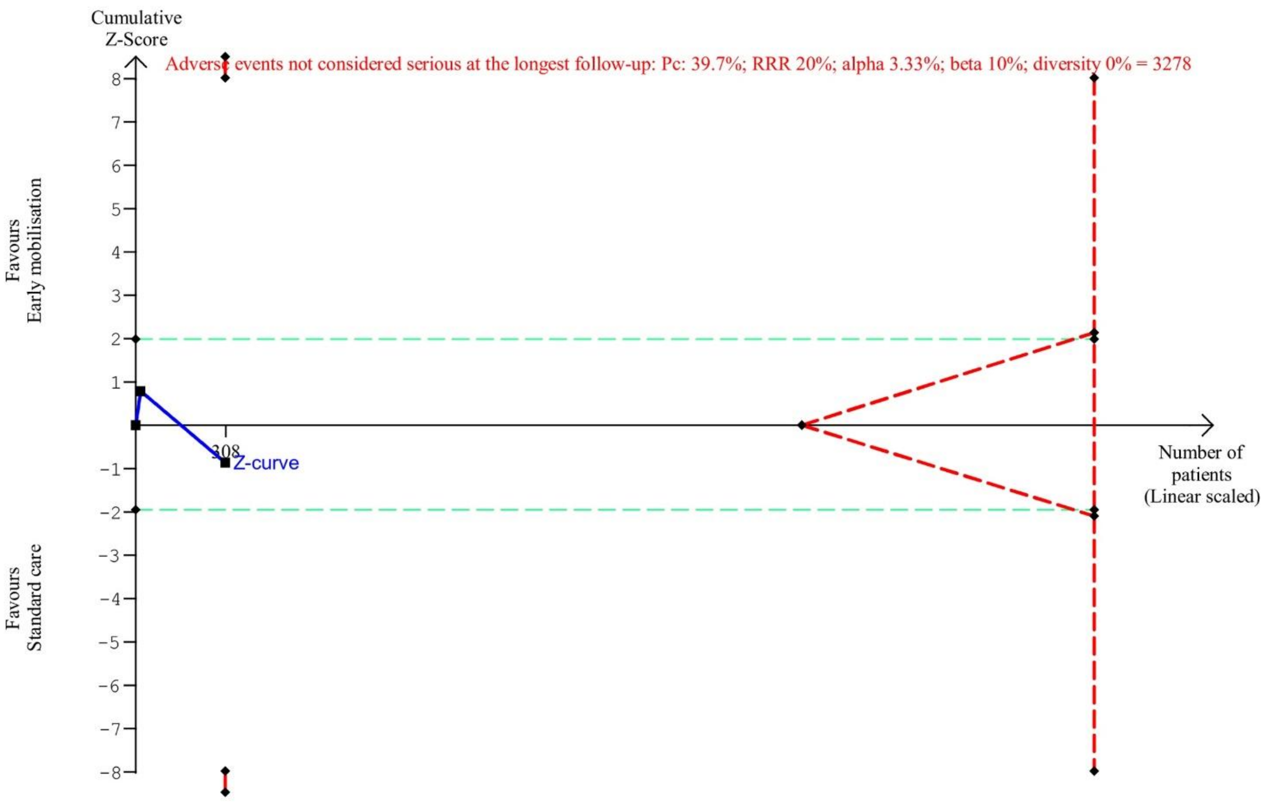

Trial Sequential Analysis of the cumulative fixed-effect meta-analysis assessing early mobilisation versus standard care on adverse events not considered serious in patients with severe acquired brain injury is based on a proportion in the control group (Pc) of 39.7%, a relative risk reduction (RRR) of 20%, an alpha of 2.5%, a beta of 10%, and diversity of 0% and equals 3499 patients. The cumulative Z-curve (blue) is far from breaking any boundaries for benefit (upper red line), harm (lower red line), or futility (the two outward sloping dashed red lines at the right).

Table 2. Number of patients with at least one individualized serious adverse event or adverse event not considered serious sorted according to total number of patients with event.

<table>
<tr><th>Serious adverse events</th><th>Number with event in early mobilisation group</th><th>Randomised in early mobilisation group</th><th>Number with event in standard care group</th><th>Randomised in standard care group</th><th>Relative risk – (95% CI)</th></tr>
<tr><td>Pneumonia [1; 2; 3]</td><td>31</td><td>176</td><td>30</td><td>170</td><td>RR 1.00 (0.76 to 1.31)</td></tr>
<tr><td>Stroke progression or recurrent stroke [1; 2]</td><td>10</td><td>157</td><td>11</td><td>151</td><td>RR 0.93 (0.59 to 1.48)</td></tr>
<tr><td>Depression [1; 3]</td><td>12</td><td>166</td><td>7</td><td>163</td><td>RR 1.27 (0.89 to 1.82)</td></tr>
<tr><td>Delirium [3]</td><td>1</td><td>19</td><td>4</td><td>19</td><td>RR 0.37 (0.06 to 2.18)</td></tr>
<tr><td>Sepsis [3]</td><td>4</td><td>19</td><td>1</td><td>19</td><td>RR 1.65 (0.95 to 2.87)</td></tr>
<tr><td>Acute myocardial infarction [1; 2; 3]</td><td>3</td><td>176</td><td>0</td><td>170</td><td>RR 4.43 (0.38 to 51.45)</td></tr>
<tr><td>Blocked tracheal tube [3]</td><td>2</td><td>19</td><td>1</td><td>19</td><td>RR 1.37 (0.58 to 3.28)</td></tr>
<tr><td>Seizures [3]</td><td>2</td><td>19</td><td>0</td><td>19</td><td>RR 9.00 (0.15 to 534.5)</td></tr>
<tr><td>Other serious infections [3]</td><td>0</td><td>19</td><td>1</td><td>19</td><td>RR 0.20 (0.00 to 13.99)</td></tr>
<tr><td>Adverse events not considered serious</td><td></td><td></td><td></td><td></td><td></td></tr>
<tr><td>Falls [1; 2; 3]</td><td>47</td><td>176</td><td>50</td><td>170</td><td>RR 0.94 (0.74 to 1.19)</td></tr>
<tr><td>Urinary tract infection [1; 2; 3]</td><td>25</td><td>176</td><td>26</td><td>170</td><td>RR 0.96 (0.71 to 1.29)</td></tr>
<tr><td>Pressure ulcer [1; 2; 3]</td><td>16</td><td>176</td><td>10</td><td>170</td><td>RR 1.23 (0.89 to 1.70)</td></tr>
<tr><td>Patients removing their nasogastric tube [3]</td><td>6</td><td>19</td><td>7</td><td>19</td><td>RR 0.89 (0.44 to 1.78)</td></tr>
<tr><td>Vomiting [3]</td><td>3</td><td>19</td><td>5</td><td>19</td><td rowspan="2">RR 0.70 (0.27 to 1.83)</td></tr>
<tr><td>Acquiring other infections [3]</td><td>3</td><td>19</td><td>5</td><td>19</td></tr>
<tr><td>Angina [1; 3]</td><td>1</td><td>29</td><td>5</td><td>26</td><td>RR 0.33 (0.05 to 1.96)</td></tr>
<tr><td>Developing paroxysmal sympathetic hyperactivity [3]</td><td>3</td><td>19</td><td>3</td><td>19</td><td>RR 1.00 (0.42 to 2.39)</td></tr>
<tr><td>Withdrawal symptoms [3]</td><td>5</td><td>19</td><td>1</td><td>19</td><td>RR 1.90 (1.12 to 3.24)</td></tr>
<tr><td>Anaemia [3]</td><td>2</td><td>19</td><td>3</td><td>19</td><td rowspan="2">RR 0.78 (0.25 to 2.39)</td></tr>
<tr><td>Diarrhoea [3]</td><td>2</td><td>19</td><td>3</td><td>19</td></tr>
<tr><td>Oral mycosis [3]</td><td>2</td><td>19</td><td>2</td><td>19</td><td>RR 1.00 (0.35 to 2.81)</td></tr>
<tr><td>Acquiring a wound</td><td>3</td><td>19</td><td>1</td><td>19</td><td>RR 1.59 (0.82 to 3.11)</td></tr>
</table>

Hypokalaemia [3]	3	19	1	19	
Hyponatremia [3]	1	19	3	19	RR 0.47 (0.08 to 2.65)
Tachycardia [3]	1	19	2	19	RR 0.65 (0.13 to 3.32)
Confusion [3]	3	19	0	19	RR 13.0 (0.2 to 723.5)
Conjunctivitis [3]	2	19	1	19	RR 1.37 (0.58 to 3.28)
bleeding from a surgical wound [3]	1	19	2	19	RR 0.65 (0.13 to 3.32)
Blocked tracheal tube [3]	0	19	2	19	
Ventriculitis [3]	0	19	2	19	
Removal of venous or arterial catheter [3]	0	19	2	19	RR 0.11 (0.00 to 6.6)
Restless [3]	0	19	2	19	
Rash [3]	0	19	2	19	
Hypertension [3]	2	19	0	19	RR 9.00 (0.15 to 534.5)
Hyperkalaemia [3]	2	19	0	19	
Hypercapnia [3]	1	19	1	19	
Hypernatraemia [3]	1	19	1	19	RR 1.00 (0.24 to 4.15)
Tongue biting [3]	1	19	1	19	
Respiratory secretion (atelectasis) [3]	1	19	0	19	
Bleeding urethra [3]	1	19	0	19	
Removal of tracheotomy [3]	1	19	0	19	
Alkalosis [3]	1	19	0	19	
Hypermagnesaemia [3]	1	19	0	19	
Hyperglycaemia [3]	1	19	0	19	
Acute tubulointerstitial nephropathy [3]	1	19	0	19	
Thrombocytosis [3]	1	19	0	19	RR 5.00 (0.07 to 349.8)
Dental fracture [3]	1	19	0	19	
Loose external ventricular drain screw [3]	1	19	0	19	
Dysfunctional arterial catheter [3]	1	19	0	19	
Distended anal sphincter [3]	1	19	0	19	
Pancreatitis [3]	1	19	0	19	
Nose bleeding [3]	1	19	0	19	
Haematoma lower extremity [3]	1	19	0	19	

Joint swelling [3]	1	19	0	19	
Desaturation [3]	0	19	1	19	RR 0.20 (0.0 to 13.99)
Hypotension [3]	0	19	1	19	
Agitated [3]	0	19	1	19	
Removal of wound dressing [3]	0	19	1	19	
Calf pain [3]	0	19	1	19	
Subcutaneous emphysema [3]	0	19	1	19	
Heart murmur [3]	0	19	1	19	
Displacement of fracture [3]	0	19	1	19	
Epidermolysis arm [3]	0	19	1	19	
Fever without origin [3]	0	19	1	19	
Increased saliva [3]	0	19	1	19	
Obstipation [3]	0	19	1	19	
Sleep apnea [3]	0	19	1	19	
Gastrointestinal bleeding [3]	0	19	1	19	

1: AVERT Trial Collaboration Group. Efficacy and safety of very early mobilisation within 24 h of stroke onset (AVERT): a randomised controlled trial. Lancet. 2015;386: 46–55. doi:10.1016/S0140-6736(15)60690-0

2: Bernhardt J, Dewey H, Thrift A, Collier J, Donnan G. A very early rehabilitation trial for stroke (AVERT): phase II safety and feasibility. Stroke. 2008;39: 390–6. doi:10.1161/STROKEAHA.107.492363

3: Riberholt CG, Olsen MH, Søndergaard CB, Gluud C, Ovesen C, Jakobsen JC, et al. Early orthostatic exercise by head-up tilt with stepping versus standard care after severe traumatic brain injury – a randomized clinical feasibility trial. Neurorehabilitation and Neural repair, Submitt May 2020.
