# Supplementary material for: Early head-up mobilisation versus standard care for patients with severe acquired brain injury: A systematic review with meta-analysis and Trial Sequential Analysis
Source: PLoS One. 2020 Aug 13;15(8):e0237136. doi: 10.1371/journal.pone.0237136 (PMC7425882; doi:10.1371/journal.pone.0237136)
Supplement: Attachment — Submitted filename: 2020_06_28_Point_by_point response.docx [file pone.0237136.s002.docx]

**Point-by-point response to reviewers	28-06-2020**

To the Editor of PLOS ONE,

We would like to thank you and the reviewers for very insightful comments and thorough revision of our manuscript. We have revised the paper accordingly and believe that it has improved considerably in this process.

Below please find our point-by point responses to the reviewers with references to changes in the revised manuscript. In addition, “track changes” are provided in the revised version of the manuscript and an unmarked version has been uploaded as well.

We have also made general corrections to the text and tables to improve the quality. Among other things, we noted that we had used risk difference on single trials with one zero event intervention group. This analysis is wrong and leads to too narrow and unrealistic confidence intervals. We have now changed these analyses into relative risks with zero event corrections. Furthermore, when we only have one trial, the P values have been checked with Chi-square tests (no zero events) or Fischer’s exact test (with zero events).

| **Editorial office comments** | **Response** | **Reference to manuscript** |
| --- | --- | --- |
| 1. Please ensure that your manuscript meets PLOS ONE's style requirements, including those for file naming. The PLOS ONE style templates can be found at <https://journals.plos.org/plosone/s/file?id=wjVg/PLOSOne_formatting_sample_main_body.pdf> and <https://journals.plos.org/plosone/s/file?id=ba62/PLOSOne_formatting_sample_title_authors_affiliations.pdf> | We thank the editorial office for pointing out these corrections. We have found the following style requirements after rereading your style guidelines:  Title page: Dept has been spelled out.  Initials for the corresponding author has been added after the e-mail address.  Changes made to all figure captions so that the legend comes directly after the figure title.  As table 3 was presented before table 2, we have changed the order of these tables.  We have gathered all the supplementary material in one file (S1 File). The in-text reference to this material will be according to the PLOS ONE guidelines (e.g. Protocol in S1 File or Table 1 in S1 File). | Page 1 |
| 2. Please include captions for your Supporting Information files at the end of your manuscript, and update any in-text citations to match accordingly. Please see our Supporting Information guidelines for more information: [http://journals.plos.org/plosone/s/supporting-information](about:blank). | After references we have added a section “Supporting information” including file names and captions for all supporting material. | Page 35 |

| **Reviewer 1 comment** | **Response** | **Reference to manuscript** |
| --- | --- | --- |
| I note that the review includes only 4 trials and 385 patients. It should be clearly acknowledged that this is a rather limited evidence base. I am concerned that it cannot really support the large number of analyses and outcomes considered, particularly in the level of detail reported, and perhaps some simplification would be appropriate; for example, to move exploratory outcomes to an appendix. | We agree with the reviewer on both comments.  In the beginning of our discussion section, we have now added the following sentence to clarify that the evidence base is limited: “It should be emphasised that the small number of trials and patients in this review only provides limited evidence.”  Furthermore, we have moved all exploratory outcomes to the S2 Table.  For two of the events (acute myocardial infarction and confusion) we originally calculated a risk difference since these events were not present in control groups of the included trials. The events were not common though and we have therefor calculated a relative risk with a correction in the control group of 0.5 events. We believe that the following is more representative for the level of evidence: “*Because there were no events in the standard care group, we calculated the RR with a 0.5 event correction. The results from the fixed-effect model yielded a RR of 4.43 for acute myocardial infarction (95% CI 0.4 to 51.5) and a RR of 13.0 for confusion (95% CI 0.2 to 723.5)”*. | Page 40  Page 33 |
| The paper is rather repetitive in places, particularly (but not only) the many “The RR of”s in the exploratory outcomes section. In general, there is no need to repeat in text what is reported in Tables or figures, and simply presenting tables of results (and forest plots), with a short text commentary, would make the paper an easier read. | We have decided to move the exploratory outcomes to the Table 2 in S1 File.  Furthermore, the TSA analysis has also now been moved to this supplement. | See S1 File |
| While I appreciate the use of TSA, I do not think it is really adding much to this paper: the evidence base is clearly too small, and the effect estimates too close to the null, for it to produce any useful conclusions. I note, for example, that in most TSA plots there is too little data even to establish the alpha spending boundaries. Perhaps the TSA analyses should be placed in an appendix, to make the paper shorter and easier to follow for readers not familiar with TSA. | We agree with the reviewer and appreciate this suggestion.  As stated above we have moved the TSA analyses to the supplemental file called **S1 File**. We believe that the TSA has an important part in avoiding type I or type II errors even in systematic reviews with low numbers, where spurious results may occur.  Even though some readers may not be familiar with the TSA, the lack of evidence are very intuitively interpreted from the illustrations. | See S1  File Fig 1 to 13 |

| **Reviewer 2 comment** | **Response** | **Reference to manuscript** |
| --- | --- | --- |
| Based on the PLOS-ONE guidelines for authors, tables should not be within the text. We suggest the authors to arrange the document as mentioned in PLOS-ONE guidelines. | Thank you for this suggestion. But when looking through the guidelines (<https://journals.plos.org/plosone/s/tables>) we found that it stated “*Place each table in your manuscript file directly after the paragraph in which it is first cited (read order). Do not submit tables in a separate file(s).*” We will be happy to comply with the reviewer’s suggestion if the editorial office so chooses. |  |
| The authors included observational data as part of the results, however, there is no information about how the risk of bias and certainty of the evidence was assessed from these sources. | We have now added the following to the section “Assessment of risk of bias in included studies”:  “*The observational studies did not undergo formal risk of bias assessment. We are aware that the observational data carry a high risk of selection bias. However, if observational studies do report harms, then in all likelihood harms occurring is at least at that level or above that level [29, 30].* | Page 13 |
| Abstract; Abstract may be improved by following the PRISMA recommendations for abstracts. | We thank the reviewer for this comment, but at the same time found it hard to stay under the recommended 300-word limit.  The abstract has now been revised according to the PRISMA guidelines and MECIR guidelines (<https://community.cochrane.org/sites/default/files/uploads/inline-files/Version%20March%202020%20Final%20Online%20version%20CLEAN_2.pdf> ).  Changes have been made to the method-section, result-section and conclusion of the abstract. | Abstract |
| Methods:  Sensitivity analysis can be described as separated section. | Thank you for the suggestion. We have now added a section called “Sensitivity analysis” and moved the relevant text from the section “Measure of treatment effect” to this section. We have also added the following sentence: “*For the primary outcome ‘mortality or poor functional outcome’ we did a sensitivity analysis for mortality and for poor functional outcome among survivors at both time points*”*.* | Page 14 |
| GRADE description may be improved, for example, to report the outcomes importance (critical, important, non important) | We have added the importance of the outcomes to the “SoF”-table and added the following in the “GRADE” section: “*Each outcome was evaluated as critical, important, or not important as recommended by the GRADE guidelines [56]*”. | Please see table 3 and page 17. |
| Non-randomized data How the observational data was assessed? | We did not undertake any assessments of the harms reported in observational studies, but reported what was reported in the observational study |  |
| Results: PLOS-ONE does not limit the number of characters in their manuscripts, however, the document feels very saturated of information that may be reported in a more structure way. If authors are using the GRADE approach, to describe the results by the importance of the outcomes might be a feasible idea. | We thank the reviewer for this comment. We have arranged the sections of the primary and secondary results so that they reflect the GRADE Summary of Findings. Furthermore, as mentioned above we have moved the TSA and the exploratory outcomes to the S1 File. |  |
| Exploratory outcomes section does not have any kind of arrangement, sorting the evidence by type of outcome or other approach might help to improve this section. | We agree with the reviewer. As mentioned in the first comment from reviewer 1, we have moved all the exploratory outcomes to the supplemental table 2 in S1 File and sorted them according to total number of patients experiencing these outcomes. | See S1 File |
| GRADE: More information needs to be provided in the GRADE explanations footnotes (Summary of Finding table). For example, We decided to downgrade by one level due to imprecision, because the effect estimate was not consistent with benefit or harm, or other kind of judgment that can help the reader to understand the reasoning behind the decision. | The reviewer makes a good point and we have elaborated on the footnotes in the GRADE explanations (Table 3). Furthermore, we have added a paragraph in the “GRADE” section on how we used the TSA to assess imprecision. It reads as follows: “*Imprecision was downgraded in GRADE (please see below) by two levels if the accrued number of participants was below 50% of the diversity-adjusted required information size (DARIS), one level if between 50% and 100% of DARIS, and no downgrade if the cumulative Z-curve reaches benefit, harm, futility, or DARIS*”. | Table 3  Page 17 |
| The quality of the figures (Forest plots and sequential analysis) was very poor, this should be improved - for some of the figures was impossible to read the results. | We sincerely apologize for this. All our graphs were approved by the PACE system used by PLOS ONE. We now hope that the new figures are of a satisfactory quality. |  |
